# Improved and semi-automated reductive β-elimination workflow for higher throughput protein *O*-glycosylation analysis

**DOI:** 10.1371/journal.pone.0210759

**Published:** 2019-01-17

**Authors:** Maximilianos Kotsias, Radoslaw P. Kozak, Richard A. Gardner, Manfred Wuhrer, Daniel I. R. Spencer

**Affiliations:** 1 Ludger Ltd, Culham Science Centre, Abingdon, Oxfordshire, United Kingdom; 2 Leiden University Medical Centre, Centre for Proteomics and Metabolomics, Leiden, Netherlands; Swiss Institute of Bioinformatics, SWITZERLAND

## Abstract

Protein *O*-glycosylation has shown to be critical for a wide range of biological processes, resulting in an increased interest in studying the alterations in *O*-glycosylation patterns of biological samples as disease biomarkers as well as for patient stratification and personalized medicine. Given the complexity of *O*-glycans, often a large number of samples have to be analysed in order to obtain conclusive results. However, most of the *O*-glycan analysis work done so far has been performed using glycoanalytical technologies that would not be suitable for the analysis of large sample sets, mainly due to limitations in sample throughput and affordability of the methods. Here we report a largely automated system for *O*-glycan analysis. We adapted reductive β-elimination release of *O*-glycans to a 96-well plate system and transferred the protocol onto a liquid handling robot. The workflow includes *O*-glycan release, purification and derivatization through permethylation followed by MALDI-TOF-MS. The method has been validated according to the ICH Q2 (R1) guidelines for the validation of analytical procedures. The semi-automated reductive β-elimination system enabled for the characterization and relative quantitation of *O*-glycans from commercially available standards. Results of the semi-automated method were in good agreement with the conventional manual in-solution method while even outperforming it in terms of repeatability. Release of *O*-glycans for 96 samples was achieved within 2.5 hours, and the automated data acquisition on MALDI-TOF-MS took less than 1 minute per sample. This largely automated workflow for *O*-glycosylation analysis showed to produce rapid, accurate and reliable data, and has the potential to be applied for *O*-glycan characterization of biological samples, biopharmaceuticals as well as for biomarker discovery.

## Introduction

Glycosylation is one of the most common protein co-translational and post-translational modifications found in all domains of life.[1] There are two main types of protein glycosylation: *N*-glycosylation, where *N*-glycans are attached to asparagines in the tripeptide consensus sequence Asn-X-Ser/Thr (where X can be any amino acid except proline), and mucin-type-*O*-glycosylation where *O*-glycans are attached to the peptide chain through a Ser or Thr residue.[2]

Much of the work for the development of new tools for glycosylation analysis has been focused on *N*-glycans. However, the study of protein *O*-glycosylation has shown to be critical for a wide range of biological processes, resulting in an increased interest in studying the alterations in *O*-glycosylation patterns of biological samples as disease biomarkers as well as for patient stratification and personalized medicine.[3–6] *O*-glycans have been reported to regulate various physiological and pathological processes such as: protecting underlying proteins as well as epithelial cell surfaces, maintaining protein conformations, and controlling active epitopes and antigenicity[7]; they modulate processes such as cell-matrix adhesion and cell-cell interactions, determine the expression and function of cell surface receptors and may be involved in growth regulation.[7,8] *O*-glycans have also been shown to play a role in infection and pathogenesis, embryogenesis, angiogenesis, development and cell death.[9–13] In cancer, *O*-glycans are altered at the earliest stages of the cellular transformation and these alterations are important in cancer initiation.[14,15] Furthermore, *O*-glycans play significant roles in the attachment, invasion and metastasis of cancer cells.[16,17] Study and research of altered *O*-glycosylation has led to the development of *O*-glycans-based biomarkers, including glycan- or glycoprotein targeted antibodies, such as CA15-3, CA19-9, CA125, and B72.3[18], highlighting, what great potential they have to serve as tools for cancer diagnosis, prognosis and treatment monitoring to aid personalized medicine.[19] *O*-glycans have also proven to be critical for the development of biopharmaceuticals.[20] Following their biosynthesis, a large number of biological drugs are post-translationally modified by the addition of *O*-linked glycans. These modifications can impact safety, efficacy, immunogenicity, solubility, protein folding and half-life of the drug.[20,21] In order to maintain consistent glycosylation patterns of biotherapeutics, efficient manufacturing processes and effective glycan characterization are required.[22]

Whilst *O*-glycosylation has been reported to be a common post-translational modification of proteins, it is demanding from an analytical point of view due to the complexity and heterogeneity of *O*-glycans.[23] *O*-glycan changes can be complex and subtle, often demanding a large number of samples to be analysed in order to obtain conclusive results.[24]

A number of techniques for the analysis of protein *O*-glycosylation are currently available. However, the release, recovery and analysis of *O*-glycans still remains very challenging, due to the absence of a universal enzyme that can remove the full range of these compounds. The enzymatic release of *O*-glycans is limited to an endo-α-*N*-acetylgalactosaminidase, an *O*-glycanase with a high specificity that only enables the removal of core 1 disaccharides (Galβ1-3GalNAcα1-) from serine or threonine.[25] Therefore, the most effective methods for universal removal of *O*-glycans from glycoproteins currently rely on chemical release.[20,25] The most common method for the chemical removal of *O*-glycans is reductive β-elimination.[2,20,26] In this method *O*-glycans are released from the glycoproteins/glycopeptides and converted into the reduced form using sodium or potassium hydroxide in conjunction with sodium or potassium borohydride which reduces the terminal monosaccharide to its alditol, preventing the oligosaccharides from degrading under the alkaline release conditions (known as peeling).[26]

Although the limitations in samples throughput have recently started to disappear, with rapid sample preparation, method integration and robotization decreasing the hands-on time required to prepare and measure glycan samples, there is still a considerable gap between *O*- and *N*-glycan glycomics in terms of throughput. Most of the *O*-glycans analysis work done so far has been performed using glycoanalytical technologies that would not be suitable for the analysis of large sample sets, mainly due to limitations in sample throughput, resolution and affordability of the methods.[27] It is clear that the development of automated workflows is necessary to reduce the time required to prepare and measure *O*-glycan samples as well as provide a system that is easy to operate, repeatable and reliable. Although reductive β-elimination is a well-established tool for the removal of *O*-glycans, its application in a high-throughput setting has not yet been achieved. This can be obtained using liquid handling robots for sample processing, cleanup and sample preparation. Automated platforms can take over many of the time-consuming and labor-intensive steps liberating the analyst to perform other tasks and providing similar performance to expert manual labor, while even outperforming manual sample preparation when large cohorts are measured.[27–29]

Here we report a largely automated workflow for higher throughput protein *O*-glycosylation analysis. We adapted reductive β-elimination release of *O*-glycans to a 96-well plate system to allow for its use with a liquid handling robot. The method is shown to work on commercially available standards. *O*-glycans were successfully released, derivatized through permethylation and analysed by MALDI-TOF-MS, which represents one of the most rapid approaches for glycan analysis. It shows intrinsic robustness, accuracy, speed and high versatility, and exhibits high throughput potential.[30–32]

## Materials and methods

### Materials

Potassium hydroxide (KOH), potassium borohydride (KBH_4_), glacial acetic acid, methanol (MeOH), super DHB matrix (2,5-dihydroxybenzoic acid and 2-hydroxy-5-methoxybenzoic acid; 9:1), mucin from bovine submaxillary glands (BSM) type I-S, fetuin from fetal bovine serum (fetuin) and mucin from porcine stomach (PSM) type II were obtained from Sigma (Dorset, UK). The 96-well release plates (4ti-0125), the PCR plates, the foil pierce seals, the semi-automatic heat sealer (HT121TS), the polypropylene collection plates and the silicone plate lids were purchased from 4titude (Surrey, UK). HT permethylation kit (LT-PERMET-96) and the cation-exchange cartridges (LC-CEX) were obtained from Ludger (Oxfordshire, UK). The VersaPlate tubes were purchased from Agilent Technologies (Stockport, UK). The ultrasonic bath (FS100B) was purchased from Decon (Hove, UK). The peptide calibration standard was purchased from Bruker Daltonics (Bremen, Germany). Samples were dried down in a Thermo Savant centrifugal evaporator from Thermo (Hampshire, UK). All automated steps in the analytical workflow described were performed using a Hamilton MICROLAB STARlet Liquid Handling Workstation from Hamilton Robotics Inc. (Bonaduz, Switzerland). The liquid handling workstation used in this study has eight independent pipetting channels and an integrated vacuum manifold system. The programs for the automated method were created using Hamilton Microlab VENUS 2 base package 4.3 software. MALDI-TOF-MS data acquisition was performed using an AutoFlex Speed instrument from Bruker Daltonics (Bremen, Germany).

### Manual release of O-glycans from glycoproteins

*O*-glycans from BSM type I-S, fetuin and PSM type II glycoproteins were released and purified following the manual in-solution reductive β-elimination procedure.[33] Permethylation was performed using the liquid handling robot. A detailed method description of manual *O*-glycan release and purification is included in the supporting information (S1 File).

### Semi-automated reductive β-elimination (96-well plate format)

The sample preparation for *O*-glycan release was adapted to a 96-well plate system to allow for its use on the liquid handling robot with the exception of few steps such as off-deck plate sealing, incubation and centrifugal evaporation.

A 96-well release plate containing the in-solution glycoprotein samples was placed on the robot deck. 40 μL of a 1 M KBH_4_ solution in 0.1 M KOH was dispensed into each reaction well containing sample on the release plate and the contents were mixed by pipetting action. The release plate was sealed with a foil pierce seal in a heat sealer and incubated in an ultrasonic bath at 60 °C for 2 hours off the robot deck. Following the incubation step, the seal was removed after brief centrifugation and the release plate was placed back on the robot deck where two aliquots of respectively 2 μL and 30 μL of glacial acetic acid were added to each reaction well to terminate the reductive β-elimination reaction.

### Automated cation-exchange (CEX) cleanup

The 96-well release plate containing the samples in solution, a 96-well plate format rack loaded with CEX resin-packed VersaPlate tubes (CEX cartridges) and a polypropylene collection plate were placed onto the robot deck to perform the CEX cleanup steps. The VersaPlate tubes used in this method are packed with 300 μL of CEX resin. The cartridges were washed using three 1 mL aliquots of water with resistivity 18.2 MΩ to neutralize the pH of the resin. Two cycles of vacuum (50 mbar below atmospheric pressure) were applied during each wash at intervals of 30 seconds to aid washing of the cartridges. Following the washing steps, the automated method moved a polypropylene collection plate into the vacuum manifold ready for sample collection. The 96-well plate format rack loaded with CEX cartridges was placed on top of the collection plate. Each sample was transferred from the 96-well release plate to a CEX cartridge. An additional 200 μL of water was added to each reaction well of the release plate followed by mixing by pipetting action, to wash out any remaining sample, and the content was transferred into the respective CEX cartridge. Purified *O*-glycans were collected by gravity in the polypropylene collection plate. 300 μL of water was added to each CEX cartridge and residual glycans were eluted by gravity. Following this step, a final vacuum cycle (100 mbar below atmospheric pressure) was applied in order to elute any remaining sample from the cartridges. After the automated CEX cleanup was completed, the polypropylene collection plate containing the samples in solution was placed in a centrifugal evaporator and the contents were dried down completely.

### Automated cleanup by MeOH evaporation

The polypropylene collection plate containing the dried samples was placed back onto the robot deck. A 1 mL aliquot of MeOH was dispensed into each well and the contents were mixed by pipetting action. Following the MeOH addition and mixing, the polypropylene collection plate containing the samples in solution was placed in a centrifugal evaporator and the contents were dried down completely.

### Automated HT permethylation and liquid-liquid extraction (LLE)

The released and purified *O*-glycans were permethylated using the LudgerTag^™^ permethylation microplate kit (LT-PERMET-96), purified by liquid-liquid extraction, dried down in a centrifugal evaporator and transferred to a PCR plate as previously described.[24] Permethylation and sample purification by liquid-liquid extraction were performed on the Hamilton MICROLAB STARlet Liquid Handling Workstation. Samples were again dried down in a centrifugal evaporator in order to be concentrated prior to MALDI-TOF-MS analysis.

### MALDI-TOF-MS

The dried and permethylated samples were re-suspended in 10 μL of 70% methanol in water. The super DHB matrix solution (1.0 μL of 5 mg/mL super DHB with 1 mM NaOH in 50% ACN) was spotted manually on a Bruker, MTP Anchor-Chip 384 well MALDI-target plate followed by an equal volume of permethylated sample, and allowed to dry. Data acquisition was performed once for each sample on the MALDI-target plate. Automated data acquisition was performed using the AutoXecute feature on the AutoFlex Speed MALDI-TOF-MS. Data acquisition and sample processing took less than 1 minute on average. For data analysis, we used Bruker Daltonics flexAnalysis software version 3.4. MALDI-TOF-MS data acquisition and processing methods are described in detail in the supporting information (S1 File).

### Glycan representation

Glycan structures were visualized using GlycoWorkBench, version 2.1.[34] Structures for glycans are depicted following the Consortium for Functional Glycomics (CFG) notation[35]: *N*-acetylglucosamine (N; blue square), fucose (F; red triangle), *N*-acetylgalactosamine (N; yellow square), galactose (H; yellow circle), *N*-acetylneuraminic acid (S; purple diamond), *N*-glycolylneuraminic acid (Sg; light-blue diamond).

## Results and discussion

We analysed the following samples: glycoprotein standards (BSM type I-S, fetuin, PSM type II) using the semi-automated method described. See supporting information (S1 File) section 2 and corresponding illustrations (Figures A and B and Tables A-C in S1 File) for details regarding *O*-glycosylation analysis of BSM type I-S, fetuin and PSM type II samples.

The methods for *O*-glycan release, glycan purification, and permethylation were validated according to the ICH Q2 (R1) guidelines for the validation of analytical procedures.[36] The validation characteristics accuracy, repeatability (intraday variation), intermediate precision (interday variation), linearity, working range, limit of quantitation and limit of detection are addressed in the following section. See supporting information (S1 File) section 3 for ICH Q2 (R1) validation details regarding specificity (Figure D in S1 File) and cross-over contamination (Figure E in S1 File). See Tables D-J in S1 File for further ICH Q2 (R1) validation details regarding accuracy, repeatability, intermediate precision, linearity, limit of quantitation and limit of detection. Ten representative *O*-glycan species, with a relative peak area (RA) above 1.80%, were chosen for the validation characteristics accuracy, repeatability (intraday variation) and intermediate precision (interday variation). Four *O*-glycan species, with a RA above 5%, were chosen for the validation characteristics linearity and working range.

### Accuracy (comparison with manual in-solution procedure)

**Fig 1:**
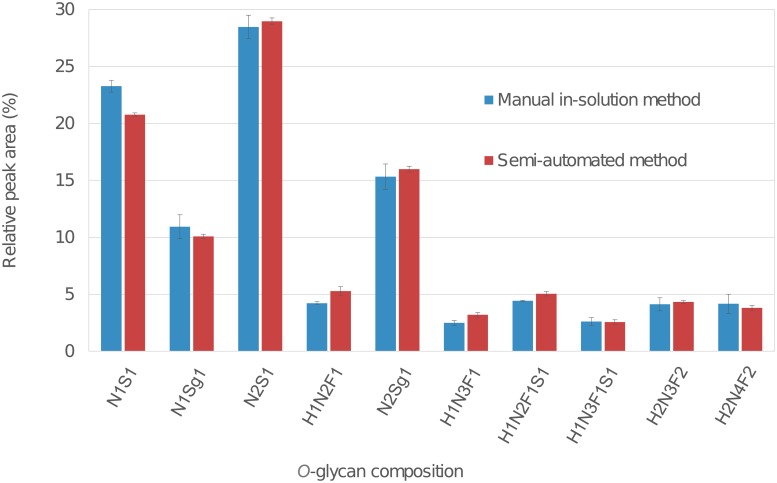
Comparison of the relative peak areas of ten representative *O*-glycans from 50 μg of BSM type I-S analysed after sample preparation using the manual in-solution method and the liquid handling robot. Blue bars depict sample preparation using the manual in-solution method. Red bars depict sample preparation using the liquid handling robot. Error bars represent standard deviation.

The accuracy of an analytical procedure expresses the closeness of agreement between the value which is accepted as a conventional true value or an accepted reference value and the value found.[36] In this study, the in-solution reductive β-elimination method was used as reference method for the development of the semi-automated method. We analysed BSM type I-S using the semi-automated reductive β-elimination method and compared the data to those obtained from the manual in-solution protocol (Fig 1). Samples were released, purified, permethylated and analysed in triplicate. The peaks from ten representative *O*-glycans were integrated and then normalized to the sum of areas. The relative areas (RAs), standard deviations (SDs), and coefficients of variation (CVs) were calculated from sample analysis using MALDI-TOF-MS (Table D in S1 File). For MALDI-TOF-MS signals with RAs above 3%, the CVs were < 6.4% compared to CVs of 20.3% for the manual in-solution method. Both spectra appear very similar, with the semi-automated reductive β-elimination method being able to produce lower CVs and with shorter processing times.

### Repeatability (intraday variation)

In order to show the precision of the procedure under the same operating conditions, glycans from 8 independent BSM type I-S samples (50 μg) were released, purified, permethylated and analysed by spotting these 8 samples singly on the MALDI-target. The areas under each peak corresponding to glycans from these spectra were integrated, normalized and the RAs, SDs, and CVs were calculated for the ten representative *O*-glycan peaks. The CVs for RAs were < 11.2% for O-glycans with RAs ≥ 2.6% (Table 1).

**Table 1:** Repeatability.

Peak no.	Glycan composition	Avg. RA (%)	SD	CV
**1**	**N1S1**	23.02	0.70	3.06
**2**	**N1Sg1**	11.34	0.74	6.56
**3**	**N2S1**	28.25	0.98	3.48
**4**	**H1N2F1**	4.39	0.24	5.40
**5**	**N2Sg1**	15.71	0.83	5.27
**6**	**H1N3F1**	2.64	0.20	7.65
**7**	**H1N2F1S1**	4.45	0.37	8.42
**8**	**H1N3F1S1**	2.49	0.24	9.69
**9**	**H2N3F2**	4.00	0.39	9.83
**10**	**H2N4F2**	3.72	0.42	11.22

Glycan composition, average relative areas (Avg. RAs), standard deviations (SDs) and coefficients of variation (CVs) for ten representative *O*-glycan structures calculated after triplicate analysis.

### Intermediate precision (interday variation)

**Fig 2:**
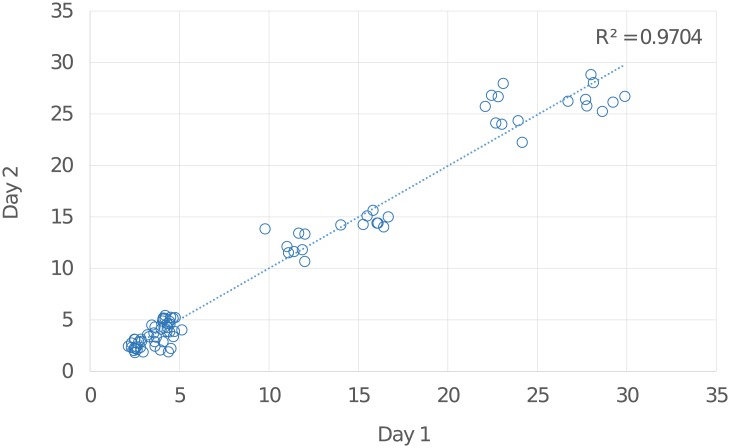
Linear regression plot comparing RA values of ten representative *O*-glycan structures. Glycans from eight independent BSM type I-S samples were released, purified, permethylated and analysed by MALDI-TOF-MS in two separate days (eight samples on day 1 versus eight samples on day 2) to assess interday variation. The R^2^ value = 0.97, which is a value close to 1 and therefore shows a good correlation and a low variation between the two data sets.

Intermediate precision or interday variation expresses within-laboratories variations[36]; different days, different analysts, different equipment etc. For this test, the same experimental set up used for the intraday variation experiment was applied to eight BSM type I-S samples (50 μg) on two separate days. Glycan areas from the spectra were integrated, normalized and the RAs, SDs and CVs were calculated for the ten representative *O*-glycan peaks. The variation between average area values for BSM type I-S *O*-glycans prepared and analysed on two different days gave CVs < 9.4% for glycans with RAs ≥ 4.5% (Table G in S1 File). A linear regression plot of the relative values from two different days gave an R^2^ value of 0.97, indicating a high level of correlation between the two data sets (Fig 2).

### Linearity

In order to evaluate the ability of the analytical procedure (within a given range) to obtain test results which are directly proportional to the amount of analyte in the sample, glycans from 200 μg, 100 μg, 50 μg, 10 μg and 5 μg of starting material of BSM type I-S glycoprotein were released, purified, permethylated and analysed in triplicate. Post release, purification and permethylation samples were each dissolved in 10 μL 70% MeOH in water. 1 μL of each sample was spotted along with an equal volume of super DHB matrix on the MALDI-target.

**Fig 3:**
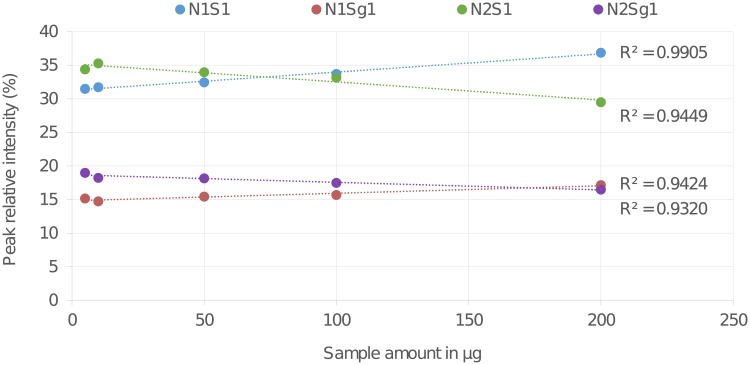
Triplicate samples of BSM type I-S glycoprotein from different starting material amounts (200 μg, 100 μg, 50 μg, 10 μg and 5 μg) released, purified and permethylated using the liquid handling robot. Relative peak intensities, extracted after MALDI-TOF-MS analysis, were plotted for four major *O*-glycan peaks. A visual evaluation of the analyte signals as function of the concentrations is shown in this linear regression plot to assess linearity as a validation parameter.

Each concentration was plotted for four major BSM type I-S *O*-glycan peaks against their relative peak intensities (RIs). The R^2^ values from the linear regression plot for the four major BSM type I-S *O*-glycan peaks were all above 0.93. A visual evaluation of the analyte signals as function of the concentrations for BSM type I-S *O*-glycans is shown in the linear regression plot to assess linearity as a validation parameter (Fig 3). See Table H in S1 File for further details regarding this validation parameter.

We believe that the negative slope shown in the concentration-signal intensity relationship of the *O*-glycan structure N_2_S_1_ and N_2_Sg_1_ is due to the stepwise degradation of these polysaccharides starting at the reducing end and removing one sugar residue at a time (peeling).[3,25,37] As previously reported, peeling can be triggered by buffer salts and other low-molecular-weight materials from glycoprotein samples.[3,37] We expect these compounds to be present in larger concentration at higher amounts of starting material. As result, peeling would increase at higher analyte amounts. In this specific case, the degradation of the terminal GlcNAc residue on the reducing end of the *O*-glycan structures N_2_S_1_ and N_2_Sg_1_ would reflect in a decrease of their relative intensities (%) and in the respective increase of the relative intensities (%) of the glycan structures N_1_S_1_ and N_1_Sg_1_. Although reductive β-elimination is known to largely prevent oligosaccharides from degradation, a complete prevention of peeling remains difficult.[25] We must point out that a limitation can be observed in the methodology where the linearity depreciates when the relative abundance of the analysed *O*-glycan species falls below a RA of 3%. This is where the range comes in. Pointing the scientists in the direction of the amount of material they should use to give the best and representative data. In this specific case, when linearity is considered to be crucial for the outcome of the analysis, the best representative data can be obtained by using an amount of starting material that ranges from 50–200 μg of glycoprotein. See supporting information (S1 File), section 3 for further details regarding the linearity of the procedure on the ten representative *O*-glycan species (Figure C and Table I in S1 File).

### Working range

The working range was derived from linearity studies, and is the interval between the lower and upper concentration (amount) of analyte in the sample for which it has been demonstrated that the analytical procedure has a suitable level of precision, accuracy and linearity.[36] An evaluation of the relative abundance of the four major *O*-glycans, and the SDs and CVs for the different amounts of glycoprotein used is shown in Table 2. From these data, it was determined that the working range was between 5 μg to 200 μg.

**Table 2:** Working range.

Amount (μg)	Glycan composition	Avg. RA (%)	SD	CV
**5**	**N1S1**	26.71	0.16	0.59
	**N1Sg1**	13.17	0.48	3.64
	**N2S1**	38.39	0.29	0.76
	**N2Sg1**	21.73	0.17	0.76
**10**	**N1S1**	26.58	2.48	9.31
	**N1Sg1**	12.66	1.18	9.33
	**N2S1**	39.69	1.46	3.69
	**N2Sg1**	21.07	2.73	12.93
**50**	**N1S1**	27.40	0.11	0.40
	**N1Sg1**	13.30	0.24	1.78
	**N2S1**	38.21	0.44	1.16
	**N2Sg1**	21.09	0.28	1.31
**100**	**N1S1**	28.57	1.30	4.54
	**N1Sg1**	13.61	0.58	4.29
	**N2S1**	37.47	1.28	3.41
	**N2Sg1**	20.35	0.60	2.97
**200**	**N1S1**	31.38	0.28	0.90
	**N1Sg1**	15.05	1.64	10.87
	**N2S1**	33.99	1.54	4.52
	**N2Sg1**	19.58	0.33	1.67

Glycan composition, average relative areas (Avg. RAs), standard deviations (SDs) and coefficients of variation (CVs) of the four major *O*-glycan structures, calculated after triplicate analysis, for the different amounts of glycoprotein used.

### Limit of quantitation and limit of detection

The quantitation limit of an analytical procedure is the lowest amount of analyte in a sample which can be quantitatively determined with suitable precision and accuracy, whereas the detection limit is described as the lowest amount of analyte in a sample which can be detected but not necessarily quantitated as an exact value.[36] Several approaches for determining the quantitation and the detection limit are possible. The approach used in this study was based on the determination of the signal-to-noise (S/N) ratios. A S/N ratio of 3:1 is generally considered acceptable for estimating the detection limit (LOD) and a S/N ratio of 10:1 is considered acceptable for quantitation purposes (LOQ). The same experimental set up used for the linearity experiment was used here where 200 μg, 100 μg, 50 μg, 10 μg and 5 μg of starting material of BSM type I-S glycoprotein was released, purified, permethylated and analysed by MALDI-TOF-MS. S/N ratios were extracted from flexAnalysis. See Table J in S1 File for a complete evaluation of this validation parameter.

## Conclusions

We have presented a largely automated *O*-glycosylation analysis workflow which was used for characterization and relative quantitation of *O*-glycans from commercially available standards (BSM type I-S, fetuin and PSM type II). Using fetuin (containing the *O*-glycans most commonly found in biopharmaceuticals)[37], BSM type I-S and PSM type II (containing complex and heterogeneous types of *O*-glycans) we demonstrate the efficacy and reliability of our system. To our knowledge, this is the first largely automated workflow for *O*-glycan release that has been adapted to a 96-well plate system to allow for its execution on a liquid handling robot, making this technology a good candidate for high throughput studies.

This work showed that the data for the identification and quantitation of *O*-glycans by MALDI-TOF-MS obtained from the semi-automated procedure were comparable to those of the conventional manual in-solution method.[2] Release of *O*-glycans for 96 samples can be performed within 2.5 h, significantly reducing the procedure timeline of the reference method (overnight incubation), without compromising the quality and integrity of the generated data (Figure F in S1 File). While providing advantages such as taking out time-consuming and labor-intensive steps, this system has showed to even outperform the current manual in-solution reductive β-elimination method in terms of repeatability, being able to produce lower CVs, for the detected structures, than previously described. The automated data acquisition using the MALDI-TOF-MS takes less than 1 minute per sample, greatly reducing the time required to measure glycan samples.

We would like to point out that although steps such as sample incubation, plate sealing and centrifugal evaporation were performed manually off the robot deck, it is possible to upgrade the Hamilton MICROLAB STARlet Liquid Handling Workstation with a heater-shaker (HHS) device, and integrated plate sealer as well as a centrifugal evaporator. MALDI-target plate spotting represents another possible source of user error as it requires a considerable degree of user concentration. This step can also be automated allowing for the automation of the whole workflow without any manual handling.[38]

In conclusion, the semi-automated reductive β-elimination system described in this article produces rapid, accurate and reliable data. We believe that this technique has the potential to be utilized for *O*-glycan characterization of biological samples, biopharmaceuticals as well as biomarker discovery.

## Supporting information

S1 File Figure A. Permethylated MALDI-TOF-MS *O*-glycans from mucin bovine submaxillary gland (BSM) type I-S. Proposed O-glycan structures, illustrated by cartoons for the ease of analysis were assigned by combining the information obtained by literature, the knowledge of glycosylation pathways and the MS/MS results from MALDI-TOF-MS data.[1] Several structures may be present in isomeric configurations. Figure B. Permethylated MALDI-TOF-MS *O*-glycans from fetuin (FET). Proposed O-glycan structures, illustrated by cartoons for the ease of analysis were assigned by combining the information obtained by literature, the knowledge of glycosylation pathways and the MS/MS results from MALDI-TOF-MS data.[2] Several structures may be present in isomeric configurations. Figure C. Triplicate samples of BSM type I-S glycoprotein from different starting material amounts (200 μg, 100 μg, 50 μg) released, purified and permethylated using the liquid handling robot. Relative peak intensities, extracted after MALDI-TOF-MS analysis, were plotted for ten representative *O*-glycan peaks. A visual evaluation of the analyte signals as function of the concentrations is shown in this linear regression plot to assess linearity as a validation parameter. A limitation can be observed in the methodology where the linearity depreciates when the relative abundance of the analysed *O*-glycan species falls below a RA of 3%.Figure D. MALDI-TOF-MS of permethylated *O*-glycans from BSM type I-S compared with water blank (negative control) analysed in parallel where the water blank underwent the same sample processing as BSM type I-S sample. Y axis is normalized to show that the negative control components do not interfere with released glycans and demonstrate specificity of the method. Figure E. MALDI-TOF-MS of permethylated *O*-glycans from BSM type I-S compared with five water blanks (negative controls), randomly dispensed into the 96-well release plate alongside BSM type I-S samples and analysed in parallel where the water blanks underwent the same sample processing as BSM type I-S samples. Y axis is normalized to show that the negative control components are negative for sample cross-over contaminations. Figure F. MALDI-TOF-MS of permethylated *O*-glycans released from BSM type I-S using 16 h incubation at 45 °C (A), 2 h sonication at 45 °C (B), 4 h sonication at 45 °C (C) and 2 h sonication at 60 °C (D). The MS signal obtained from each condition was point compared. Signal intensity and the areas of the separated *O*-glycan species from BSM type I-S were used for the comparisons. Following these experiments, the incubation at 60 °C for 2 h in an ultrasonic bath was chosen as the standard operating condition as compromise between structural coverage and incubation time. Figure G. Fragment ion spectra of precursor *m/z* 936.49, [M + Na]^+^ from BSM type I-S *O*-glycans. Figure H. Fragment ion spectra of precursor *m/z* 1331.69, [M + Na]^+^ from BSM type I-S *O*-glycans. Figure I. Fragment ion spectra of precursor *m/z* 1488.77, [M + Na]^+^ from BSM type I-S *O*-glycans. Table A. Peak numbers, *m/z* values, glycan compositions and RAs of permethylated MALDI-TOF-MS *O*-glycans from mucin from bovine submaxillary gland (BSM) type I-S. Hexose (H), HexNAc (N), Fucose (F), *N*-acetylneuraminic acid (S), *N*-glycolylneuraminic acid (Sg). Table B. Peak numbers, *m/z* values, glycan compositions and RAs of permethylated MALDI-TOF-MS *O*-glycans from fetuin. Hexose (H), HexNAc (N), Fucose (F), *N*-acetylneuraminic acid (S), *N*-glycolylneuraminic acid (Sg). Table C. Peak numbers, *m/z* values, glycan compositions and RAs of permethylated MALDI-TOF-MS *O*-glycans from PSM type II. Hexose (H), HexNAc (N), Fucose (F), *N*-acetylneuraminic acid (S), *N*-glycolylneuraminic acid (Sg). Table D. Peak numbers, glycan composition, relative areas (RAs), standard deviations (SDs) and coefficients of variation (CVs) for ten representative *O*-glycan structures released, purified, permethylated and analysed in triplicate by MALDI-TOF-MS. Table E. Peak numbers, glycan composition, relative areas (RAs), standard deviations (SDs) and coefficients of variation (CVs) for BSM type I-S detected *O*-glycan structures released, purified, permethylated and analysed in triplicate by MALDI-TOF-MS. Table F. Peak numbers, glycan composition, relative areas (RAs), standard deviations (SDs) and coefficients of variation (CVs) for BSM type I-S detected *O*-glycan structures released, purified, permethylated and analysed by MALDI-TOF-MS. Glycans from eight independent BSM type I-S samples were released, purified, permethylated and analysed by MALDI-TOF-MS to assess intraday variation. Table G. Peak numbers, glycan composition, relative areas (RAs), standard deviations (SDs) and coefficients of variation (CVs) for ten representative *O*-glycan structures released, purified, permethylated and analysed by MALDI-TOF-MS. Glycans from eight independent BSM type I-S samples were released, purified, permethylated and analysed by MALDI-TOF-MS in two separate days (eight samples on day 1 versus eight samples on day 2) to assess interday variation. Table H. Sample concentration, glycan composition, relative intensities (RIs), standard deviations (SDs) and coefficients of variation (CVs) for four major *O*-glycan structures. Triplicate samples of BSM type I-S from different starting concentration were released, purified, permethylated and analysed by MALDI-TOF-MS to assess the linearity of the procedure. Table I. Sample concentration, glycan composition, intensities and relative intensities (RIs), for ten representative *O*-glycan structures. Triplicate samples of BSM type I-S from different starting concentration were released, purified, permethylated and analysed by MALDI-TOF-MS to assess at what range of the procedure the relationship between sample amount and relative intensity of each of the glycan species is represented by a linear function. Table J. Table with signal to noise (S/N) ratios and relative areas (RAs) of permethylated BSM type I-S *O*-glycans released by semi-automated reductive β-elimination. Samples with different concentrations (200 μg, 100 μg, 50 μg, 10 μg, and 5 μg) were spotted onto the MALDI target. Within the assessed working range, S/N ratios are not significantly affected by the increase and/or decrease in sample concentration. S/N ratios in green are acceptable for LOQ, white are acceptable for LOD and not quantifiable and grey are not acceptable for quantitation or for detection. Note: LOQ = 10:1 and LOD = 3:1 of S/N ratio. Table K. Peak numbers, glycan composition, areas, relative areas (RAs), standard deviations (SDs) and coefficients of variation (CVs) for ten representative *O*-glycan structures released, purified, permethylated and analysed in triplicate by MALDI-TOF-MS. Table L. Peak numbers, glycan composition, areas, relative areas (RAs), standard deviations (SDs) and coefficients of variation (CVs) for BSM type I-S detected *O*-glycan structures released, purified, permethylated and analysed in triplicate by MALDI-TOF-MS. Table M. Peak numbers, glycan composition, areas, relative areas (RAs), standard deviations (SDs) and coefficients of variation (CVs) for ten representative BSM type I-S *O*-glycan structures released, purified, permethylated and analysed by MALDI-TOF-MS. Glycans from eight independent BSM type I-S samples were released, purified, permethylated and analysed by MALDI-TOF-MS to assess intraday variation. Table N. Peak numbers, glycan composition, areas, relative areas (RAs), standard deviations (SDs) and coefficients of variation (CVs) for BSM type I-S detected *O*-glycan structures released, purified, permethylated and analysed by MALDI-TOF-MS. Glycans from eight independent BSM type I-S samples were released, purified, permethylated and analysed by MALDI-TOF-MS to assess intraday variation. Table O. Peak numbers, glycan composition, areas, relative areas (RAs), standard deviations (SDs) and coefficients of variation (CVs) for ten representative O-glycan structures released, purified, permethylated and analysed by MALDI-TOF-MS. Glycans from eight independent BSM type I-S samples were released, purified, permethylated and analysed by MALDI-TOF-MS in two separate days (eight samples on day 1 versus eight samples on day 2) to assess interday variation. Table P. Sample concentration, glycan composition, intensities and relative intensities (RIs), for ten representative *O*-glycan structures. Triplicate samples of BSM type I-S from different starting concentration were released, purified, permethylated and analysed by MALDI-TOF-MS to assess at what range of the procedure the relationship between sample amount and relative intensity of each of the glycan species is represented by a linear function.
(DOCX)
