# Supplementary material for: Improved and semi-automated reductive β-elimination workflow for higher throughput protein O-glycosylation analysis
Source: PLoS One. 2019 Jan 17;14(1):e0210759. doi: 10.1371/journal.pone.0210759 (PMC6336230; doi:10.1371/journal.pone.0210759)
Supplement: S1 File — Figure A. Permethylated MALDI-TOF-MS O-glycans from mucin bovine submaxillary gland (BSM) type I-S. Proposed O-glycan structures, illustrated by cartoons for the ease of analysis were assigned by combining the information obtained by literature, the knowledge of glycosylation pathways and the MS/MS results from MALDI-TOF-MS data.[1] Several structures may be present in isomeric configurations. Figure B. Permethylated MALDI-TOF-MS O-glycans from fetuin (FET). Proposed O-glycan structures, illustrated by cartoons for the ease of analysis were assigned by combining the information obtained by literature, the knowledge of glycosylation pathways and the MS/MS results from MALDI-TOF-MS data.[2] Several structures may be present in isomeric configurations. Figure C. Triplicate samples of BSM type I-S glycoprotein from different starting material amounts (200 μg, 100 μg, 50 μg) released, purified and permethylated using the liquid handling robot. Relative peak intensities, extracted after MALDI-TOF-MS analysis, were plotted for ten representative O-glycan peaks. A visual evaluation of the analyte signals as function of the concentrations is shown in this linear regression plot to assess linearity as a validation parameter. A limitation can be observed in the methodology where the linearity depreciates when the relative abundance of the analysed O-glycan species falls below a RA of 3%.Figure D. MALDI-TOF-MS of permethylated O-glycans from BSM type I-S compared with water blank (negative control) analysed in parallel where the water blank underwent the same sample processing as BSM type I-S sample. Y axis is normalized to show that the negative control components do not interfere with released glycans and demonstrate specificity of the method. Figure E. MALDI-TOF-MS of permethylated O-glycans from BSM type I-S compared with five water blanks (negative controls), randomly dispensed into the 96-well release plate alongside BSM type I-S samples and analysed in parallel where the [file pone.0210759.s001.docx]

**Improved and semi-automated reductive β-elimination workflow for higher throughput protein *O*-glycosylation analysis**

Maximilianos Kotsias^1*^, Radoslaw P. Kozak^1¶^, Richard A. Gardner^1¶^, Manfred Wuhrer^2^, Daniel I. R. Spencer^1^

^1^Ludger Ltd, Culham Science Centre, Abingdon, Oxfordshire, UK

^2^Leiden University Medical Centre, Centre for Proteomics and Metabolomics, Leiden, Netherlands

***Corresponding author**

E-mail: [maximilianos.kotsias@ludger.com](mailto:maximilianos.kotsias@ludger.com)

**Supporting information**

**Table of contents:**

**Experimental protocols – Section 1** **3**

**1.1** Materials 3

**1.2** Manual in-solution *O*-glycan release 3

**1.3** Manual cation-exchange (CEX) cleanup 3

**1.4** Manual cleanup by MeOH evaporation3

**1.5** Sample preparation for automated and high-throughput (HT) permethylation 4

**1.6** MALDI-TOF-MS 4

**Results – Section 25**

**Figure A**5

**Table A**6

**Figure B**7

**Table B**7

**Table C**8

**ICH Q2 (R1) Validation – Section 39**

**Tables D-I**11

**Figure C**19

**Table J**20

**Figures D, E**21

**Additional experimental data – Section 423**

**Figures F-I**23

**Additional data** **– Section 5** (data points behind all statistical studies)**27**

**Tables K-P**28

**References38**

**Experimental protocols – Section 1**

**1.1 Materials**

The 1.5 mL Eppendorf® Safe-Lock microcentrifuge tubes, the Parafilm® M sealing film, potassium hydroxide (KOH), potassium borohydrade (KBH_4_), glacial acetic acid, methanol (MeOH), super DHB matrix (2,5-dihydroxybenzoic acid and 2-hydroxy-5-methoxybenzoic acid; 9:1) and mucin from bovine submaxillary glands (BSM) type I-S, were obtained from Sigma (Dorset, UK). The PCR plates, the foil pierce seals, the semi-automatic heat sealer (HT121TS), the polypropylene collection plates and the silicone plate lids were purchased from 4titude (Surrey, UK). HT permethylation kit (LT-PERMET-96) and the cation-exchange cartridges (LC-CEX) were obtained from Ludger (Oxfordshire, UK). The VersaPlate tubes were purchased from Agilent Technologies (Stockport, UK). The ultrasonic bath (FS100B) was purchased from Decon (Hove, UK). The peptide calibration standard was purchased from Bruker Daltonics (Bremen, Germany). Samples were dried down in a Thermo Savant centrifugal evaporator from Thermo (Hampshire, UK). All automated steps in the analytical workflow described were performed using a Hamilton MICROLAB STARlet Liquid Handling Workstation from Hamilton Robotics Inc. (Bonaduz, Switzerland). MALDI-TOF-MS data acquisition was performed using AutoFlex Speed instrument from Bruker Daltonics (Bremen, Germany).

**1.2 Manual in-solution *O*-glycan release**

50 µg of BSM type I-S glycoprotein sample was dispensed into a 1.5 mL Eppendorf® Safe-Lock microcentrifuge tube. 40 μL of a 1M KBH_4_ solution in 0.1M KOH was dispensed into each tube containing sample and the contents were mixed by pipetting action. The tubes were locked, sealed with a layer of Parafilm® M sealing film and incubated in an ultrasonic bath at 60°C for 2 hours. Following the incubation step, the seal was removed after brief centrifugation and two aliquots of respectively 2 μL and 30 μL of glacial acetic acid were added to each tube to terminate the reductive β-elimination reaction.

**1.3 Manual cation-exchange (CEX) cleanup**

The VersaPlate used in this method are packed with 300 µL of CEX resin and loaded on a 96-well plate format rack. The cartridges were washed using three 1 mL aliquots of water with resistivity 18.2 MΩ to neutralize the pH of the resin. The water eluted by gravity after each wash was discarded. Following the washing steps, each sample was transferred from the 1.5 mL Eppendorf® tubes to a CEX cartridge. An additional 200 µL of water was added to each tube followed by mixing by pipetting action, to wash out any remaining sample, and the content was transferred into the respective CEX cartridge. Purified O-glycans were collected by gravity in a polypropylene collection plate. 300 µL of water was added to each CEX cartridge and residual glycans were eluted by gravity. Following this step, a Gilson PIPETMAN® P1000 was used to apply pressure on the top of each cartridge in order to elute any remaining samples from the cartridges. After the CEX cleanup was completed, the polypropylene collection plate containing the samples in solution was placed in a centrifugal evaporator and the contents were dried down completely.

**1.4 Manual cleanup by MeOH evaporation**

A 1 mL aliquot of MeOH was dispensed into each well of the polypropylene collection plate containing the dried samples and the contents were mixed by pipetting action using a Gilson PIPETMAN® Multichannel P200. Following the MeOH addition and mixing, the polypropylene collection plate containing the samples in solution was placed in a centrifugal evaporator and the contents were dried down completely.

**1.5 Sample preparation for automated and high-throughput (HT) permethylation**

All steps for sample preparation were automated using a liquid handling robot with the exception of few steps such as off-deck plate sealing, centrifugal evaporation and sample spotting on the MALDI target plate.

**1.6 MALDI-TOF-MS**

Automated data acquisition was performed using the AutoXecute feature on the AutoFlex Speed MALDI-TOF-MS instrument from Bruker with a Smartbeam-II laser and system speed of 2 kHz for MS and 200 Hz for MS/MS. The instrument was used in high resolution reflectron positive (RP) mode. FlexControl 3.4 software build 119 from Bruker Daltonics was used for acquiring data for all mass spectral measurements. A window of *m/z*  300-2000 was used for BSM type I-S and fetuin glycoprotein samples and *m/z* 300-3500 was used for PSM type II glycoprotein samples data acquisition. 20,000 laser shots were accumulated at a laser frequency of 1000 Hz using a random walk style pattern with 50 shots per spot. Peptide calibration standard from Bruker Daltonics was used to calibrate the instrument. Data acquisition for each sample measurement/acquisition using MALDI-TOF-MS was performed in triplicate and averaged to provide relative peak intensities and relative peak areas. Bruker Daltonics flexAnalysis software version 3.4 was utilized for data representation of [M+Na]^+^ permethylated glycans.

**Results – Section 2**


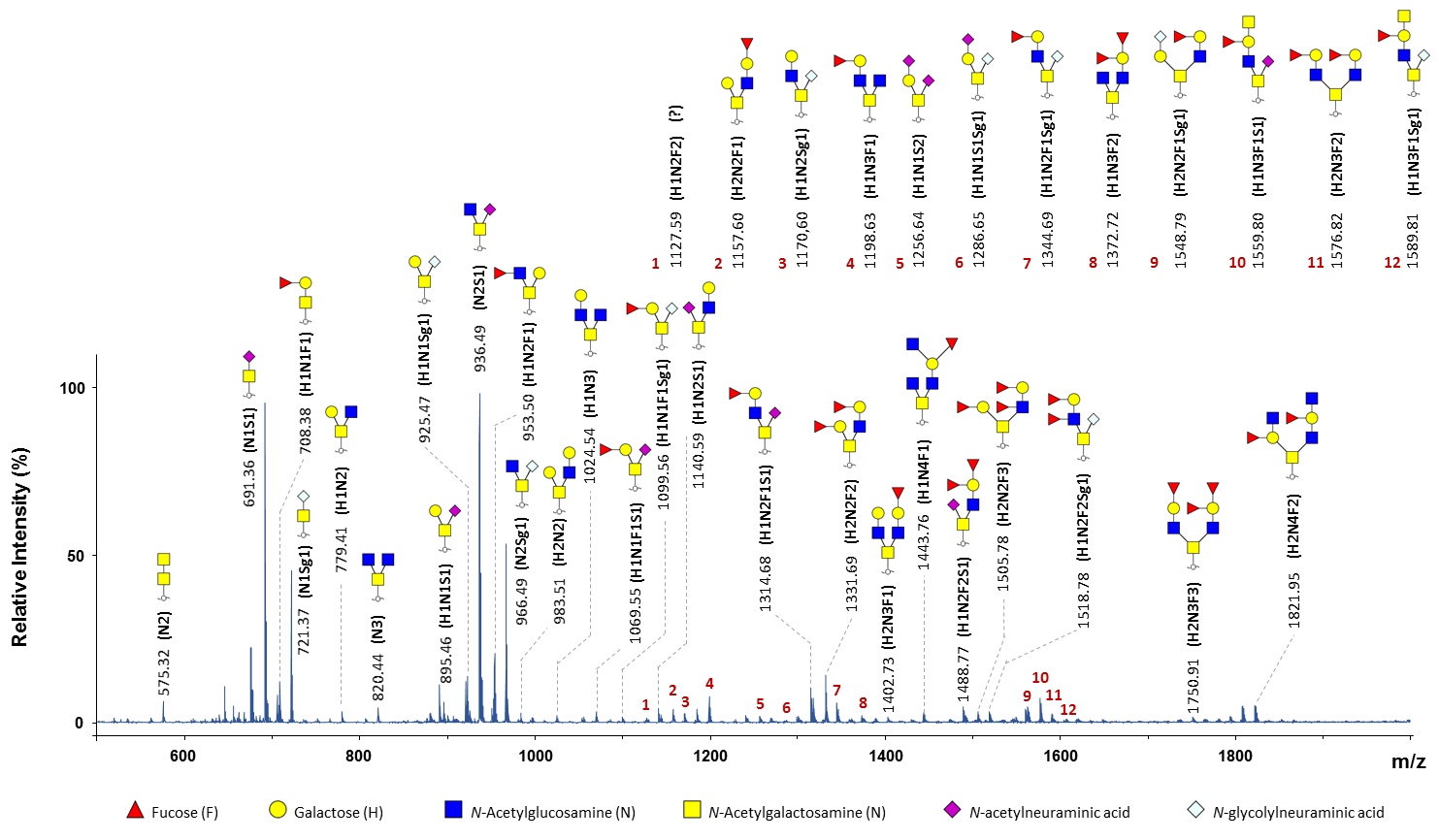


**Figure A.**

| MALDI-TOF-MS data of permethylated *O*-glycans from BSM type I-S glycoprotein released by semi-automated reductive β-elimination | | | | | | | | | | | | | | | | |
| --- | --- | --- | --- | --- | --- | --- | --- | --- | --- | --- | --- | --- | --- | --- | --- | --- |
| Peak no. | ***m/z*** | **Composition** | | | | | **RA (%)** |  | **Peak no.** | ***m/z*** | **Composition** | | | | | **RA (%)** |
|  |  | **H** | **N** | **F** | **S** | **Sg** |  |  |  |  | **H** | **N** | **F** | **S** | **Sg** |  |
| 1 | **575.32** | - | 2 | - | - | - | **0.81** |  | 21 | **1228.64** | 1 | 2 | 2 | - | - | **0.23** |
| 2 | **691.36** | - | 1 | - | 1 | - | **14.65** |  | 22 | **1256.64** | 1 | 1 | - | 2 | - | **0.57** |
| 3 | **708.38** | 1 | 1 | 1 | - | - | **1.62** |  | 23 | **1286.65** | 1 | 1 | - | 1 | 1 | **0.21** |
| 4 | **721.37** | - | 1 | - | - | 1 | **7.11** |  | 24 | **1314.68** | 1 | 2 | 1 | 1 | - | **3.56** |
| 5 | **779.41** | 1 | 2 | - | - | - | **0.57** |  | 25 | **1331.69** | 2 | 2 | 2 | - | - | **4.71** |
| 6 | **820.44** | - | 3 | - | - | - | **0.80** |  | 26 | **1344.69** | 2 | 2 | - | 1 | - | **2.08** |
| 7 | **895.46** | 1 | 1 | - | 1 | - | **1.15** |  | 27 | **1361.70** | 3 | 2 | 1 | - | - | **0.36** |
| 8 | **925.47** | 1 | 1 | - | - | 1 | **0.62** |  | 28 | **1372.72** | 1 | 3 | 2 | - | - | **0.71** |
| 9 | **936.49** | - | 2 | - | 1 | - | **20.43** |  | 29 | **1402.73** | 2 | 3 | 1 | - | - | **0.58** |
| 10 | **953.50** | 1 | 2 | 1 | - | - | **3.73** |  | 30 | **1443.76** | 1 | 4 | 1 | - | - | **1.03** |
| 11 | **966.50** | - | 2 | - | - | 1 | **11.28** |  | 31 | **1488.77** | 1 | 2 | 2 | 1 | - | **1.93** |
| 12 | **983.51** | 2 | 2 | - | - | - | **0.54** |  | 32 | **1505.78** | 2 | 2 | 3 | - | - | **1.23** |
| 13 | **1024.54** | 1 | 3 | - | - | - | **0.45** |  | 33 | **1518.78** | 1 | 2 | 2 | - | 1 | **1.32** |
| 14 | **1069.55** | 1 | 1 | 1 | 1 | - | **0.85** |  | 34 | **1548.79** | 2 | 2 | 1 | - | 1 | **0.60** |
| 15 | **1099.56** | 1 | 1 | 1 | - | 1 | **0.43** |  | 35 | **1559.80** | 1 | 3 | 1 | 1 | - | **1.81** |
| 16 | **1127.59** | 1 | 2 | 2 | - | - | **0.37** |  | 36 | **1576.82** | 2 | 3 | 2 | - | - | **3.05** |
| 17 | **1140.59** | 1 | 2 | - | 1 | - | **1.17** |  | 37 | **1589.81** | 1 | 3 | 1 | - | 1 | **1.14** |
| 18 | **1157.60** | 2 | 2 | 1 | - | - | **1.10** |  | 38 | **1750.91** | 2 | 3 | 3 | - | - | **0.73** |
| 19 | **1170.60** | 1 | 2 | - | - | 1 | **0.77** |  | 39 | **1793.91** | 3 | 3 | - | 1 | - | **0.77** |
| 20 | **1198.63** | 1 | 3 | 1 | - | - | **2.25** |  | 40 | **1821.95** | 2 | 4 | 2 | - | - | **2.69** |

**Table A.**


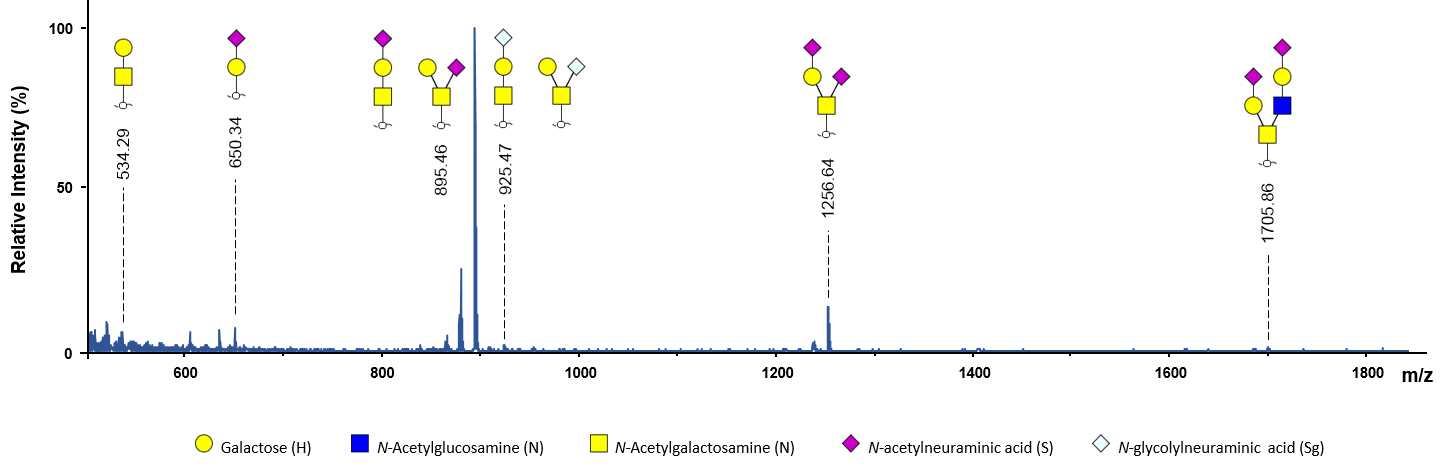


**(H2N2S2)**

**(H1N1S2)**

**(H1N1Sg1)**

**(H1N1S1)**

**(H1S1)**

**(H1N1)**

**Figure B.**

| MALDI-TOF-MS data of permethylated *O*-glycans from fetuin glycoprotein released by semi-automated reductive β-elimination | | | | | | | | | | | | | | | | |
| --- | --- | --- | --- | --- | --- | --- | --- | --- | --- | --- | --- | --- | --- | --- | --- | --- |
| Peak no. | ***m/z*** | **Composition** | | | | | **RA (%)** |  | **Peak no.** | ***m/z*** | **Composition** | | | | | **RA (%)** |
|  |  | **H** | **N** | **F** | **S** | **Sg** |  |  |  |  | **H** | **N** | **F** | **S** | **Sg** |  |
| 1 | **534.29** | 1 | 1 | - | - | - | **7.03** |  | 4 | **925.47** | 1 | 1 | - | - | 1 | **0.69** |
| 2 | **650.34** | 1 | - | - | 1 | - | **6.93** |  | 5 | **1256.64** | 1 | 1 | - | 2 | - | **12.83** |
| 3 | **895.46** | 1 | 1 | - | 1 | - | **71.42** |  | 6 | **1705.86** | 2 | 2 | - | 2 | - | **1.09** |

**Table B.**

| MALDI-TOF-MS data of permethylated *O*-glycans from PSM type II glycoprotein released by semi-automated reductive β-elimination | | | | | | | | | | | | | | | | |
| --- | --- | --- | --- | --- | --- | --- | --- | --- | --- | --- | --- | --- | --- | --- | --- | --- |
| Peak no. | ***m/z*** | **Composition** | | | | | **RA (%)** |  | **Peak no.** | ***m/z*** | **Composition** | | | | | **RA (%)** |
|  |  | **H** | **N** | **F** | **S** | **Sg** |  |  |  |  | **H** | **N** | **F** | **S** | **Sg** |  |
| 1 | **534.29** | 1 | 1 | - | - | - | **3.48** |  | 36 | **1518.78** | 1 | 2 | 2 | - | 1 | **0.19** |
| 2 | **575.32** | - | 2 | - | - | - | **0.14** |  | 37 | **1535.79** | 3 | 2 | 2 | - | - | **0.13** |
| 3 | **691.36** | - | 1 | - | 1 | - | **0.22** |  | 38 | **1576.82** | 2 | 3 | 2 | - | - | **0.28** |
| 4 | **708.38** | 1 | 1 | 1 | - | - | **10.13** |  | 39 | **1589.81** | 1 | 3 | 1 | - | 1 | **0.12** |
| 5 | **738.39** | 2 | 1 | - | - | - | **0.34** |  | 40 | **1606.83** | 3 | 3 | 1 | - | - | **0.35** |
| 6 | **749.40** | - | 2 | 1 | - | - | **0.42** |  | 41 | **1647.86** | 2 | 4 | 1 | - | - | **2.43** |
| 7 | **779.41** | 1 | 2 | - | - | - | **29.90** |  | 42 | **1675.85** | 1 | 2 | 1 | 2 | - | **0.04** |
| 8 | **895.46** | 1 | 1 | - | 1 | - | **1.44** |  | 43 | **1677.87** | 3 | 4 | - | - | - | **0.33** |
| 9 | **912.48** | 2 | 1 | 1 | - | - | **0.18** |  | 44 | **1692.87** | 2 | 2 | 2 | 1 | - | **0.16** |
| 10 | **925.47** | 1 | 1 | - | - | 1 | **0.33** |  | 45 | **1718.89** | 2 | 5 | - | - | - | **0.19** |
| 11 | **936.49** | - | 2 | - | 1 | - | **1.03** |  | 46 | **1763.90** | 2 | 3 | 1 | 1 | - | **0.07** |
| 12 | **953.50** | 1 | 2 | 1 | - | - | **4.47** |  | 47 | **1780.92** | 3 | 3 | 2 | - | - | **0.50** |
| 13 | **966.50** | - | 2 | - | - | 1 | **0.29** |  | 48 | **1793.91** | 3 | 3 | - | 1 | - | **0.04** |
| 14 | **983.51** | 2 | 2 | - | - | - | **2.21** |  | 49 | **1821.95** | 2 | 4 | 2 | - | - | **0.19** |
| 15 | **1024.54** | 1 | 3 | - | - | - | **5.05** |  | 50 | **1851.96** | 3 | 4 | 1 | - | - | **0.51** |
| 16 | **1069.55** | 1 | 1 | 1 | 1 | - | **0.27** |  | 51 | **1881.97** | 4 | 4 | - | - | - | **0.13** |
| 17 | **1127.59** | 1 | 2 | 2 | - | - | **0.18** |  | 52 | **1922.99** | 3 | 5 | - | - | - | **0.34** |
| 18 | **1140.59** | 1 | 2 | - | 1 | - | **1.01** |  | 53 | **1968.00** | 3 | 3 | 1 | 1 | - | **0.23** |
| 19 | **1157.60** | 2 | 2 | 1 | - | - | **2.85** |  | 54 | **2009.03** | 2 | 4 | 1 | 1 | - | **0.12** |
| 20 | **1198.63** | 1 | 3 | 1 | - | - | **0.68** |  | 55 | **2026.05** | 3 | 4 | 2 | - | - | **0.76** |
| 21 | **1228.64** | 1 | 2 | 2 | - | - | **3.21** |  | 56 | **2056.06** | 4 | 4 | 1 | - | - | **0.15** |
| 22 | **1256.64** | 1 | 1 | - | 2 | - | **0.23** |  | 57 | **2097.08** | 3 | 5 | 1 | - | - | **0.44** |
| 23 | **1269.67** | 1 | 4 | - | - | - | **1.38** |  | 58 | **2168.12** | 3 | 6 | - | - | - | **0.30** |
| 24 | **1314.68** | 1 | 2 | 1 | 1 | - | **0.35** |  | 59 | **2230.15** | 4 | 4 | 2 | - | - | **0.27** |
| 25 | **1316.66** | 1 | 1 | - | - | 2 | **1.03** |  | 60 | **2271.17** | 3 | 5 | 2 | - | - | **0.19** |
| 26 | **1331.69** | 2 | 2 | 2 | - | - | **2.52** |  | 61 | **2342.21** | 3 | 6 | 1 | - | - | **0.16** |
| 27 | **1344.69** | 2 | 2 | - | 1 | - | **0.15** |  | 62 | **2404.23** | 4 | 4 | 3 | - | - | **0.50** |
| 28 | **1361.70** | 3 | 2 | 1 | - | - | **0.18** |  | 63 | **2475.27** | 4 | 5 | 2 | - | - | **0.34** |
| 29 | **1372.72** | 1 | 3 | 2 | - | - | **0.13** |  | 64 | **2516.26** | 3 | 3 | - | 3 | - | **0.13** |
| 30 | **1374.70** | 2 | 2 | - | - | 1 | **0.23** |  | 65 | **2591.32** | 4 | 4 | 2 | 1 | - | **0.07** |
| 31 | **1402.73** | 2 | 3 | 1 | - | - | **7.80** |  | 66 | **2604.31** | 4 | 4 | - | 2 | - | **0.08** |
| 32 | **1443.76** | 1 | 4 | 1 | - | - | **0.39** |  | 67 | **2720.40** | 4 | 6 | 2 | - | - | **0.11** |
| 33 | **1447.74** | 2 | 1 | 2 | 1 | - | **0.42** |  | 68 | **2853.46** | 5 | 5 | 3 | - | - | **0.06** |
| 34 | **1473.77** | 2 | 4 | - | - | - | **6.90** |  | 69 | **3098.59** | 5 | 6 | 3 | - | - | **0.06** |
| 35 | **1505.78** | 2 | 2 | 3 | - | - | **0.17** |  |  |  |  |  |  |  |  |  |

**Table C.**

**ICH Q2 (R1) Validation – Section 3**

The methods for *O*-glycan release, purification and permethylation were validated according to the ICH Q2 (R1) guidelines for the validation of analytical procedures. The following validation characteristics; accuracy, repeatability (intraday variation), intermediate precision (interday variation), linearity, working range, limit of quantitation, limit of detection and specificity are included in this paragraph.

- 1. **Accuracy (A – ten representative *O*-glycans), (B – detected *O*-glycans).** The accuracy of an analytical procedure expresses the closeness of agreement between the value which is accepted as a conventional true value or an accepted reference value and the value found.[3] In this study, the in-solution reductive β-elimination method was used as reference standard for the development of the semi-automated method. We analysed BSM type I-S using the semi-automated reductive β-elimination method and compared the data to those obtained from the manual in-solution protocol. Samples were released, purified, permethylated and analysed in triplicate. The peaks from 10 major *O*-glycans were integrated and then normalized to the sum of areas. The relative areas (RAs), standard deviations (SDs), and coefficients of variation (CVs) were calculated from sample analysis using MALDI-TOF-MS (Table D). For MALDI-TOF-MS signals with RAs above 3%, the CVs were <6.41% compared to CVs of 20.32% for the manual in-solution method. Both spectra appear very similar, with the semi-automated reductive β-elimination method being able to produce lower CVs and faster processing times.
  2. **Repeatability (detected *O*-glycans).** In order to show the precision of the procedure under the same operating conditions, glycans from eight independent BSM type I-S samples (50 μg) were released, purified, permethylated and analysed by spotting these eight samples singly on the MALDI-target. The areas under each peak corresponding to glycans from these spectra were integrated, normalized and the RAs, SDs, and CVs were calculated for all detected *O*-glycan peaks (Table F).
  3. **Intermediate precision (interday variation).** Intermediate precision or interday variation expresses within-laboratories variations[3]; different days, different analysts, different equipment etc. For this test, the same experimental set up used for the intraday variation experiment (repeatability, manuscript) was applied to 8 BSM type I-S samples (50 µg) on two separate days. Glycan areas from the spectra were integrated, normalized and the RAs, SDs and CVs were calculated for the 10 major *O*-glycan peaks. The variation between average area values for BSM type I-S *O*-glycans prepared and analysed on two different days gave CVs <9.42% for glycans with RAs ≥4.50% (Table E). A linear regression plot of the relative values from two different days gave an R^2^ value of 0.97, indicating a high level of correlation between the two data sets (Fig 2, manuscript).
  4. **Linearity.** Linearity of an analytical procedure is its ability (within a given range) to obtain test results which are directly proportional to the concentration (amount) of analyte in the sample.[3] A linear relationship should be evaluated across the range of the analytical procedure. In order to evaluate linearity, glycans from 200 µg, 100 µg, 50 µg, 10 µg and 5 µg of starting material of BSM type I-S glycoprotein were released, purified, permethylated and analysed in triplicate. Post release, purification and permethylation samples were each dissolved in 10 µL 70% MeOH in water. 1 µL of each sample was spotted along with an equal volume of super DHB matrix on the MALDI-target. Each concentration was plotted for four major (Table H) and ten representative (Table I) BSM type I-S *O*-glycan peaks against their relative peak intensities (RIs). The R^2^ values from the linear regression plot for the four major BSM type I-S *O*-glycan peaks were all above 0.93 (Fig 3, manuscript). An evaluation of the R^2^ values from the linear regression plot for the ten representative BSM type I-S *O*-glycan peaks is shown in Figure C.
  5. **Limit of Quantitation and Limit of Detection.** The quantitation limit of an analytical procedure is the lowest amount of analyte in a sample which can be quantitatively determined with suitable precision and accuracy, whereas the detection limit is described as the lowest amount of analyte in a sample which can be detected but not necessarily quantitated as an exact value.[3] Several approaches for determining the quantitation and the detection limit are possible. The approach used in this study was based on the determination of the signal-to-noise (S/N) ratios. A S/N ratio of 3:1 is generally considered acceptable for estimating the detection limit (LOD) and a S/N ratio of 10:1 is considered acceptable for quantitation purposes (LOQ). The same experimental set up used for the linearity experiment was used where 200 µg, 100 µg, 50 µg, 10 µg and 5 µg of starting material of BSM type I-S glycoprotein was released, purified, permethylated and analysed by MALDI-TOF-MS. S/N ratios were extracted from flexAnalysis. See Table G for a complete evaluation of this validation parameter.
  6. **Specificity.** Specificity is the ability to assess unequivocally the analyte in the presence of components which may be expected to be present.[3] Typically these might include impurities, degradants, matrix, etc. Specificity was demonstrated by verifying that the negative control (water blank) components do not interfere with released *O*-glycans after glycan release, purification and permethylation. See Figure C for comparison spectra of permethylated BSM type I-S *O*-glycans and water blank.
  7. **Cross-over contamination.** To evaluate the presence of cross contamination, the analyte and a blank sample are successively analysed. If a signal is detected in the negative control at the same *m/z* value of the analyte, the cross contamination is significant. No cross contamination was demonstrated by randomly dispensing five negative controls (water blanks) into the 96-well release plate alongside BSM type I-S samples. All the negative control components analysed in parallel, where the water blanks underwent the same sample processing as BSM type I-S samples, showed to be negative for sample cross-over contaminations (Figure D).

**Working range has been described in the manuscript under the results and discussion section.**

- 1. **Accuracy (A – ten representative *O*-glycans)**

| MALDI-TOF-MS data of permethylated *O*-glycans released by manual in-solution reductive β-elimination | | | | | | | |  | MALDI-TOF-MS data of permethylated *O*-glycans released by semi-automated reductive β-elimination | | | | | | | |
| --- | --- | --- | --- | --- | --- | --- | --- | --- | --- | --- | --- | --- | --- | --- | --- | --- |
| Peak no. | **Glycan composition** | **Replicate A** | **Replicate B** | **Replicate C** | **Data** | | |  | **Peak no.** | **Glycan composition** | **Replicate A** | **Replicate B** | **Replicate C** | **Data** | | |
|  |  | **RA (%)** | **RA (%)** | **RA (%)** | **Avg. RA (%)** | **SD** | **CV** |  |  |  | **RA (%)** | **RA (%)** | **RA (%)** | **Avg. RA (%)** | **SD** | **CV** |
| 1 | **N1S1** | 22.67 | 23.63 | 23.49 | **23.26** | 0.52 | **2.22** |  | **1** | **N1S1** | 20.64 | 20.95 | 20.70 | **20.76** | 0.16 | **0.79** |
| 2 | **N1Sg1** | 10.85 | 9.92 | 12.02 | **10.93** | 1.05 | **9.63** |  | **2** | **N1Sg1** | 10.24 | 10.13 | 9.86 | **10.08** | 0.20 | **1.96** |
| 3 | **N2S1** | 27.62 | 29.61 | 28.16 | **28.46** | 1.03 | **3.63** |  | **3** | **N2S1** | 28.62 | 29.04 | 29.20 | **28.95** | 0.30 | **1.03** |
| 4 | **H1N2F1** | 4.34 | 4.07 | 4.24 | **4.22** | 0.14 | **3.24** |  | **4** | **H1N2F1** | 4.89 | 5.31 | 5.65 | **5.28** | 0.38 | **7.27** |
| 5 | **N2Sg1** | 15.43 | 14.14 | 16.38 | **15.32** | 1.12 | **7.33** |  | **5** | **N2Sg1** | 16.16 | 16.09 | 15.70 | **15.98** | 0.25 | **1.55** |
| 6 | **H1N3F1** | 2.74 | 2.42 | 2.31 | **2.49** | 0.23 | **9.11** |  | **6** | **H1N3F1** | 3.04 | 3.12 | 3.43 | **3.20** | 0.20 | **6.40** |
| 7 | **H1N2F1S1** | 4.38 | 4.41 | 4.48 | **4.42** | 0.05 | **1.15** |  | **7** | **H1N2F1S1** | 5.15 | 4.82 | 5.18 | **5.05** | 0.20 | **3.97** |
| 8 | **H1N3F1S1** | 2.69 | 2.91 | 2.22 | **2.61** | 0.35 | **13.44** |  | **8** | **H1N3F1S1** | 2.79 | 2.50 | 2.41 | **2.57** | 0.20 | **7.81** |
| 9 | **H2N3F2** | 4.64 | 4.21 | 3.51 | **4.12** | 0.57 | **13.85** |  | **9** | **H2N3F2** | 4.47 | 4.22 | 4.30 | **4.33** | 0.13 | **2.93** |
| 10 | **H2N4F2** | 4.63 | 4.68 | 3.19 | **4.17** | 0.85 | **20.32** |  | **10** | **H2N4F2** | 4.01 | 3.82 | 3.58 | **3.80** | 0.21 | **5.64** |

**Table D.**

- 1. **Accuracy (B – detected *O*-glycans)**

| MALDI-TOF-MS data of permethylated *O*-glycans released by manual in-solution reductive β-elimination | | | | | | | |  | MALDI-TOF-MS data of permethylated *O*-glycans released by semi-automated reductive β-elimination | | | | | | | |
| --- | --- | --- | --- | --- | --- | --- | --- | --- | --- | --- | --- | --- | --- | --- | --- | --- |
| Peak no. | **Glycan composition** | **Replicate A** | **Replicate B** | **Replicate C** | **Data** | | |  | **Peak no.** | **Glycan composition** | **Replicate A** | **Replicate B** | **Replicate C** | **Data** | | |
|  |  | **RA (%)** | **RA (%)** | **RA (%)** | **Avg. RA (%)** | **SD** | **CV** |  |  |  | **RA (%)** | **RA (%)** | **RA (%)** | **Avg. RA (%)** | **SD** | **CV** |
| 1 | **N2** | 0.66 | 0.46 | 0.46 | **0.52** | 0.12 | **22.01** |  | 1 | **N2** | 0.66 | 0.85 | 0.93 | **0.81** | 0.14 | **17.33** |
| 2 | **N1S1** | 16.31 | 17.54 | 17.40 | **17.08** | 0.67 | **3.94** |  | 2 | **N1S1** | 14.58 | 14.82 | 14.54 | **14.65** | 1.15 | **1.02** |
| 3 | **H1N1F1** | 1.55 | 1.33 | 1.47 | **1.45** | 0.11 | **7.72** |  | 3 | **H1N1F1** | 1.50 | 1.67 | 1.69 | **1.62** | 0.10 | **6.41** |
| 4 | **N1Sg1** | 7.81 | 7.36 | 8.90 | **8.02** | 0.79 | **9.87** |  | 4 | **N1Sg1** | 7.24 | 7.17 | 6.93 | **7.11** | 0.16 | **2.29** |
| 5 | **H1N2** | 0.54 | 0.39 | 0.41 | **0.45** | 0.08 | **18.04** |  | 5 | **H1N2** | 0.52 | 0.59 | 0.60 | **0.57** | 0.04 | **7.23** |
| 6 | **N3** | 0.80 | 0.59 | 0.58 | **0.66** | 0.13 | **19.34** |  | 6 | **N3** | 0.71 | 0.85 | 0.83 | **0.80** | 0.08 | **9.70** |
| 7 | **H1N1S1** | 1.09 | 1.33 | 1.31 | **1.24** | 0.13 | **10.53** |  | 7 | **H1N1S1** | 1.16 | 1.17 | 1.13 | **1.15** | 0.02 | **1.73** |
| 8 | **H1N1Sg1** | 0.54 | 0.49 | 0.59 | **0.54** | 0.05 | **9.07** |  | 8 | **H1N1Sg1** | 0.62 | 0.61 | 0.61 | **0.62** | 0.01 | **0.99** |
| 9 | **N2S1** | 19.87 | 21.98 | 20.86 | **20.90** | 1.06 | **5.07** |  | 9 | **N2S1** | 20.23 | 20.54 | 20.52 | **20.43** | 0.18 | **0.87** |
| 10 | **H1N2F1** | 3.12 | 3.02 | 3.14 | **3.09** | 0.06 | **2.09** |  | 10 | **H1N2F1** | 3.45 | 3.76 | 3.97 | **3.73** | 0.26 | **7.01** |
| 11 | **N2Sg1** | 11.10 | 10.50 | 12.13 | **11.25** | 0.83 | **7.35** |  | 11 | **N2Sg1** | 11.42 | 11.38 | 11.03 | **11.28** | 0.21 | **1.89** |
| 12 | **H2N2** | 0.47 | 0.43 | 0.38 | **0.43** | 0.05 | **11.21** |  | 12 | **H2N2** | 0.50 | 0.58 | 0.52 | **0.54** | 0.04 | **8.09** |
| 13 | **H1N3** | 0.38 | 0.32 | 0.35 | **0.35** | 0.03 | **8.22** |  | 13 | **H1N3** | 0.43 | 0.50 | 0.42 | **0.45** | 0.04 | **9.52** |
| 14 | **H1N1F1S1** | 0.74 | 1.02 | 1.00 | **0.92** | 0.16 | **17.28** |  | 14 | **H1N1F1S1** | 0.87 | 0.82 | 0.85 | **0.85** | 0.02 | **2.67** |
| 15 | **H1N1F1Sg1** | 0.39 | 0.47 | 0.50 | **0.45** | 0.05 | **12.04** |  | 15 | **H1N1F1Sg1** | 0.44 | 0.41 | 0.42 | **0.43** | 0.02 | **4.31** |
| 16 | **H1N2F2** | 0.27 | 0.25 | 0.25 | **0.26** | 0.01 | **4.60** |  | 16 | **H1N2F2** | 0.30 | 0.38 | 0.43 | **0.37** | 0.06 | **17.08** |
| 17 | **H1N2S1** | 1.04 | 1.17 | 1.12 | **1.11** | 0.07 | **6.33** |  | 17 | **H1N2S1** | 1.20 | 1.16 | 1.14 | **1.17** | 0.03 | **2.49** |
| 18 | **H2N2F1** | 0.94 | 0.87 | 0.87 | **0.89** | 0.04 | **4.25** |  | 18 | **H2N2F1** | 1.09 | 1.06 | 1.14 | **1.10** | 0.04 | **3.38** |
| 19 | **H1N2Sg1** | 0.70 | 0.64 | 0.69 | **0.68** | 0.04 | **5.18** |  | 19 | **H1N2Sg1** | 0.78 | 0.76 | 0.78 | **0.77** | 0.01 | **1.58** |
| 20 | **H1N3F1** | 1.97 | 1.80 | 1.71 | **1.83** | 0.14 | **7.40** |  | 20 | **H1N3F1** | 2.15 | 2.21 | 2.41 | **2.25** | 0.14 | **6.06** |
| 21 | **H1N2F2** | 0.24 | 0.20 | 0.16 | **0.20** | 0.04 | **21.41** |  | 21 | **H1N2F2** | 0.22 | 0.25 | 0.23 | **0.23** | 0.01 | **6.02** |
| 22 | **H1N1S2** | 0.73 | 0.90 | 0.80 | **0.81** | 0.08 | **10.18** |  | 22 | **H1N1S2** | 0.63 | 0.54 | 0.54 | **0.57** | 0.05 | **9.48** |
| 23 | **H1N1S1Sg1** | 0.24 | 0.31 | 0.25 | **0.27** | 0.04 | **13.66** |  | 23 | **H1N1S1Sg1** | 0.22 | 0.22 | 0.18 | **0.21** | 0.02 | **9.51** |
| 24 | **H1N2F1S1** | 3.15 | 3.27 | 3.32 | **3.25** | 0.09 | **2.66** |  | 24 | **H1N2F1S1** | 3.64 | 3.41 | 3.64 | **3.56** | 0.13 | **3.74** |
| 25 | **H2N2F2** | 4.04 | 3.78 | 4.07 | **3.96** | 0.16 | **4.03** |  | 25 | **H2N2F2** | 4.69 | 4.50 | 4.94 | **4.71** | 0.22 | **4.63** |
| 26 | **H2N2S1** | 1.91 | 1.78 | 1.98 | **1.89** | 0.10 | **5.44** |  | 26 | **H2N2S1** | 2.13 | 2.02 | 2.09 | **2.08** | 0.05 | **2.53** |
| 27 | **H3N2F1** | 0.45 | 0.35 | 0.26 | **0.35** | 0.10 | **18.29** |  | 27 | **H3N2F1** | 0.34 | 0.37 | 0.37 | **0.36** | 0.01 | **4.15** |
| 28 | **H1N3F2** | 0.61 | 0.46 | 0.48 | **0.52** | 0.08 | **15.92** |  | 28 | **H1N3F2** | 0.66 | 0.71 | 0.75 | **0.71** | 0.05 | **6.35** |
| 29 | **H2N3F1** | 0.60 | 0.51 | 0.43 | **0.51** | 0.09 | **16.61** |  | 29 | **H2N3F1** | 0.62 | 0.58 | 0.54 | **0.58** | 0.04 | **6.18** |
| 30 | **H1N4F1** | 1.15 | 0.78 | 0.73 | **0.89** | 0.23 | **25.62** |  | 30 | **H1N4F1** | 1.02 | 1.05 | 1.01 | **1.03** | 0.02 | **1.86** |
| 31 | **H1N2F2S1** | 1.63 | 1.64 | 1.66 | **1.64** | 0.01 | **0.82** |  | 31 | **H1N2F2S1** | 1.95 | 1.88 | 1.97 | **1.93** | 0.05 | **2.61** |
| 32 | **H2N2F3** | 1.11 | 1.03 | 1.06 | **1.06** | 0.04 | **3.75** |  | 32 | **H2N2F3** | 1.26 | 1.17 | 1.26 | **1.23** | 0.05 | **4.02** |
| 33 | **H1N2F2Sg1** | 1.24 | 1.07 | 1.18 | **1.16** | 0.09 | **7.36** |  | 33 | **H1N2F2Sg1** | 1.34 | 1.29 | 1.34 | **1.32** | 0.03 | **2.44** |
| 34 | **H2N2F1Sg1** | 0.80 | 0.54 | 0.51 | **0.62** | 0.16 | **26.35** |  | 34 | **H2N2F1Sg1** | 0.62 | 0.62 | 0.56 | **0.60** | 0.03 | **5.81** |
| 35 | **H1N3F1S1** | 1.94 | 2.16 | 1.64 | **1.91** | 0.26 | **13.42** |  | 35 | **H1N3F1S1** | 1.97 | 1.77 | 1.69 | **1.81** | 0.14 | **8.01** |
| 36 | **H2N3F2** | 3.34 | 3.13 | 2.60 | **3.02** | 0.38 | **12.59** |  | 36 | **H2N3F2** | 3.16 | 2.98 | 3.02 | **3.05** | 0.09 | **2.97** |
| 37 | **H1N3F1Sg1** | 1.28 | 1.23 | 1.04 | **1.18** | 0.13 | **10.88** |  | 37 | **H1N3F1Sg1** | 1.25 | 1.14 | 1.04 | **1.14** | 0.10 | **9.19** |
| 38 | **H2N3F3** | 0.83 | 0.77 | 0.67 | **0.76** | 0.08 | **10.65** |  | 38 | **H2N3F3** | 0.78 | 0.71 | 0.70 | **0.73** | 0.05 | **6.27** |
| 39 | **H3N3S1** | 1.09 | 0.67 | 0.70 | **0.82** | 0.23 | **28.28** |  | 39 | **H3N3S1** | 0.81 | 0.78 | 0.71 | **0.77** | 0.05 | **6.65** |
| 40 | **H2N4F2** | 3.33 | 3.47 | 2.36 | **3.05** | 0.60 | **19.79** |  | 40 | **H2N4F2** | 2.83 | 2.70 | 2.52 | **2.69** | 0.16 | **5.93** |

**Table E.**

- 1. **Repeatability (detected *O*-glycans)**

| Peak no. | Glycan composition | Replicate A | Replicate B | Replicate C | Replicate D | Replicate E | Replicate F | Replicate G | Replicate H |  | Avg. RA (%) | SD | CV |
| --- | --- | --- | --- | --- | --- | --- | --- | --- | --- | --- | --- | --- | --- |
|  |  | **RA (%)** | **RA (%)** | **RA (%)** | **RA (%)** | **RA (%)** | **RA (%)** | **RA (%)** | **RA (%)** |  |  |  |  |
| 1 | **N2** | 0.86 | 0.72 | 0.64 | 0.71 | 1.09 | 1.09 | 0.78 | 0.79 |  | **0.84** | 0.17 | **20.19** |
| 2 | **N1S1** | 16.31 | 16.92 | 16.93 | 15.88 | 17.55 | 16.97 | 16.22 | 16.88 |  | **16.71** | 0.53 | **3.19** |
| 3 | **H1N1F1** | 1.52 | 1.53 | 1.49 | 1.50 | 1.65 | 1.74 | 1.59 | 1.51 |  | **1.57** | 0.09 | **5.75** |
| 4 | **N1Sg1** | 8.47 | 8.90 | 7.16 | 7.97 | 8.71 | 8.41 | 7.87 | 8.35 |  | **8.23** | 0.55 | **6.71** |
| 5 | **H1N2** | 0.55 | 0.52 | 0.49 | 0.50 | 0.66 | 0.63 | 0.54 | 0.58 |  | **0.56** | 0.06 | **10.90** |
| 6 | **N3** | 0.75 | 0.76 | 0.63 | 0.68 | 0.86 | 0.85 | 0.76 | 0.82 |  | **0.76** | 0.08 | **10.35** |
| 7 | **H1N1S1** | 1.19 | 1.30 | 1.18 | 1.16 | 1.13 | 1.15 | 1.21 | 1.15 |  | **1.18** | 0.06 | **4.66** |
| 8 | **H1N1Sg1** | 0.57 | 0.60 | 0.46 | 0.52 | 0.71 | 0.66 | 0.57 | 0.71 |  | **0.60** | 0.09 | **14.73** |
| 9 | **N2S1** | 20.14 | 21.25 | 21.91 | 20.12 | 20.44 | 18.95 | 19.86 | 21.44 |  | **20.51** | 0.96 | **4.70** |
| 10 | **H1N2F1** | 3.37 | 3.03 | 3.07 | 3.09 | 3.04 | 3.19 | 3.40 | 3.27 |  | **3.18** | 0.15 | **4.66** |
| 11 | **N2Sg1** | 11.65 | 12.18 | 10.27 | 11.57 | 11.49 | 10.82 | 11.07 | 12.22 |  | **11.41** | 0.66 | **5.82** |
| 12 | **H2N2** | 0.47 | 0.46 | 0.41 | 0.40 | 0.62 | 0.61 | 0.42 | 0.56 |  | **0.49** | 0.09 | **18.07** |
| 13 | **H1N3** | 0.42 | 0.34 | 0.37 | 0.41 | 0.50 | 0.46 | 0.43 | 0.46 |  | **0.42** | 0.05 | **12.07** |
| 14 | **H1N1F1S1** | 0.79 | 0.80 | 0.95 | 0.84 | 0.70 | 0.75 | 0.85 | 0.76 |  | **0.80** | 0.08 | **9.42** |
| 15 | **H1N1F1Sg1** | 0.43 | 0.46 | 0.41 | 0.42 | 0.39 | 0.39 | 0.41 | 0.41 |  | **0.41** | 0.02 | **5.65** |
| 16 | **H1N2F2** | 0.33 | 0.27 | 0.28 | 0.32 | 0.22 | 0.34 | 0.33 | 0.25 |  | **0.29** | 0.04 | **14.42** |
| 17 | **H1N2S1** | 0.11 | 1.18 | 1.21 | 1.17 | 1.05 | 1.08 | 1.11 | 1.11 |  | **1.13** | 0.05 | **4.72** |
| 18 | **H2N2F1** | 0.97 | 0.88 | 0.94 | 0.98 | 0.91 | 1.05 | 1.03 | 0.97 |  | **0.97** | 0.06 | **5.87** |
| 19 | **H1N2Sg1** | 0.74 | 0.75 | 0.65 | 0.76 | 0.82 | 0.81 | 0.73 | 0.76 |  | **0.75** | 0.05 | **6.81** |
| 20 | **H1N3F1** | 1.97 | 1.72 | 1.90 | 2.02 | 1.81 | 2.04 | 2.03 | 1.81 |  | **1.91** | 0.12 | **6.33** |
| 21 | **H1N2F2** | 0.20 | 0.18 | 0.21 | 0.20 | 0.26 | 0.26 | 0.21 | 0.23 |  | **0.22** | 0.03 | **13.91** |
| 22 | **H1N1S2** | 0.54 | 0.63 | 0.72 | 0.63 | 0.55 | 0.57 | 0.60 | 0.60 |  | **0.61** | 0.06 | **9.63** |
| 23 | **H1N1S1Sg1** | 0.21 | 0.21 | 0.28 | 0.23 | 0.24 | 0.21 | 0.22 | 0.21 |  | **0.23** | 0.02 | **9.95** |
| 24 | **H1N2F1S1** | 3.22 | 3.06 | 3.41 | 3.68 | 2.95 | 3.15 | 3.37 | 2.97 |  | **3.23** | 0.25 | **7.76** |
| 25 | **H2N2F2** | 4.23 | 3.58 | 4.21 | 4.69 | 3.45 | 4.17 | 4.29 | 3.54 |  | **4.02** | 0.44 | **10.98** |
| 26 | **H2N2S1** | 1.98 | 1.92 | 1.81 | 2.18 | 1.84 | 2.01 | 2.04 | 1.85 |  | **1.95** | 0.13 | **6.41** |
| 27 | **H3N2F1** | 0.34 | 0.29 | 0.38 | 0.34 | 0.43 | 0.42 | 0.35 | 0.38 |  | **0.37** | 0.05 | **12.44** |
| 28 | **H1N3F2** | 0.55 | 0.47 | 0.51 | 0.60 | 0.54 | 0.64 | 0.61 | 0.53 |  | **0.55** | 0.06 | **10.25** |
| 29 | **H2N3F1** | 0.54 | 0.50 | 0.59 | 0.56 | 0.54 | 0.60 | 0.53 | 0.49 |  | **0.54** | 0.04 | **7.40** |
| 30 | **H1N4F1** | 0.94 | 0.82 | 0.87 | 0.93 | 0.94 | 1.05 | 1.01 | 0.82 |  | **0.92** | 0.08 | **8.89** |
| 31 | **H1N2F2S1** | 1.59 | 1.55 | 1.63 | 1.77 | 1.42 | 1.59 | 1.75 | 1.53 |  | **1.60** | 0.11 | **7.13** |
| 32 | **H2N2F3** | 1.09 | 0.94 | 1.12 | 1.22 | 0.95 | 1.10 | 1.23 | 1.01 |  | **1.08** | 0.11 | **10.28** |
| 33 | **H1N2F2Sg1** | 1.17 | 1.07 | 1.08 | 1.22 | 1.07 | 1.16 | 1.28 | 1.10 |  | **1.14** | 0.08 | **6.60** |
| 34 | **H2N2F1Sg1** | 0.56 | 0.50 | 0.52 | 0.55 | 0.79 | 0.77 | 0.67 | 0.68 |  | **0.63** | 0.11 | **17.95** |
| 35 | **H1N3F1S1** | 1.78 | 1.85 | 2.17 | 1.89 | 1.67 | 1.75 | 1.81 | 1.56 |  | **1.81** | 0.18 | **9.86** |
| 36 | **H2N3F2** | 2.96 | 2.64 | 3.20 | 3.09 | 2.63 | 3.07 | 3.09 | 2.52 |  | **2.90** | 0.26 | **8.97** |
| 37 | **H1N3F1Sg1** | 1.15 | 1.20 | 1.22 | 1.22 | 1.14 | 1.11 | 1.18 | 1.03 |  | **1.15** | 0.07 | **5.72** |
| 38 | **H2N3F3** | 0.71 | 0.65 | 0.75 | 0.72 | 0.70 | 0.75 | 0.81 | 0.70 |  | **0.72** | 0.05 | **6.57** |
| 39 | **H3N3S1** | 0.80 | 0.70 | 0.63 | 0.68 | 1.15 | 1.03 | 0.90 | 1.07 |  | **0.87** | 0.20 | **22.75** |
| 40 | **H2N4F2** | 2.85 | 2.67 | 3.31 | 2.60 | 2.38 | 2.62 | 2.84 | 2.35 |  | **2.70** | 0.31 | **11.32** |

**Table F.**

- 1. **Intermediate precision (interday variation)**

| Day 1 | Peak no. | Glycan composition | Replicate A | Replicate B | Replicate C | Replicate D | Replicate E | Replicate F | Replicate G | Replicate H |  | Avg. RA (%) | SD | CV |
| --- | --- | --- | --- | --- | --- | --- | --- | --- | --- | --- | --- | --- | --- | --- |
|  |  |  | **RA (%)** | **RA (%)** | **RA (%)** | **RA (%)** | **RA (%)** | **RA (%)** | **RA (%)** | **RA (%)** |  |  |  |  |
|  | 1 | **N1S1** | 22.43 | 22.80 | 23.08 | 22.08 | 24.14 | 23.91 | 22.66 | 23.01 |  | **24.13** | 1.79 | **7.43** |
|  | 2 | **N1Sg1** | 11.65 | 12.00 | 9.77 | 11.08 | 11.99 | 11.85 | 11.00 | 11.38 |  | **11.82** | 1.04 | **8.81** |
|  | 3 | **N2S1** | 27.69 | 28.62 | 29.88 | 27.98 | 28.12 | 26.70 | 27.76 | 29.22 |  | **27.47** | 1.33 | **4.83** |
|  | 4 | **H1N2F1** | 4.63 | 4.08 | 4.19 | 4.30 | 4.18 | 4.49 | 4.75 | 4.46 |  | **4.74** | 0.45 | **9.41** |
|  | 5 | **N2Sg1** | 16.02 | 16.40 | 14.00 | 16.09 | 15.81 | 15.25 | 15.47 | 16.65 |  | **15.18** | 0.88 | **5.77** |
|  | 6 | **H1N3F1** | 2.71 | 2.31 | 2.59 | 2.81 | 2.50 | 2.87 | 2.84 | 2.47 |  | **2.73** | 0.28 | **10.10** |
|  | 7 | **H1N2F1S1** | 4.43 | 4.12 | 4.65 | 5.12 | 4.06 | 4.43 | 4.71 | 4.05 |  | **4.36** | 0.49 | **11.33** |
|  | 8 | **H1N3F1S1** | 2.45 | 2.50 | 2.96 | 2.62 | 2.30 | 2.47 | 2.53 | 2.13 |  | **2.32** | 0.28 | **12.28** |
|  | 9 | **H2N3F2** | 4.07 | 3.56 | 4.37 | 4.29 | 3.62 | 4.32 | 4.32 | 3.44 |  | **3.88** | 0.70 | **18.16** |
|  | 10 | **H2N4F2** | 3.92 | 3.60 | 4.51 | 3.62 | 3.28 | 3.69 | 3.96 | 3.20 |  | **3.36** | 0.67 | **20.04** |

| Day 2 | Peak no. | Glycan composition | Replicate A | Replicate B | Replicate C | Replicate D | Replicate E | Replicate F | Replicate G | Replicate H |
| --- | --- | --- | --- | --- | --- | --- | --- | --- | --- | --- |
|  |  |  | **RA (%)** | **RA (%)** | **RA (%)** | **RA (%)** | **RA (%)** | **RA (%)** | **RA (%)** | **RA (%)** |
|  | 1 | **N1S1** | 26.83 | 26.69 | 27.97 | 25.75 | 22.26 | 24.36 | 24.13 | 24.02 |
|  | 2 | **N1Sg1** | 13.44 | 13.35 | 13.85 | 11.53 | 10.69 | 11.82 | 12.13 | 11.64 |
|  | 3 | **N2S1** | 26.43 | 25.25 | 26.72 | 28.84 | 28.05 | 26.25 | 25.79 | 26.16 |
|  | 4 | **H1N2F1** | 5.17 | 5.18 | 5.44 | 4.65 | 5.16 | 5.26 | 5.23 | 4.60 |
|  | 5 | **N2Sg1** | 14.42 | 14.04 | 14.23 | 14.41 | 15.65 | 14.27 | 15.11 | 15.02 |
|  | 6 | **H1N3F1** | 2.87 | 2.77 | 2.39 | 2.33 | 3.10 | 2.92 | 3.13 | 3.14 |
|  | 7 | **H1N2F1S1** | 3.84 | 4.25 | 3.39 | 4.04 | 5.09 | 4.86 | 3.89 | 4.89 |
|  | 8 | **H1N3F1S1** | 2.04 | 1.84 | 1.88 | 2.13 | 2.33 | 2.28 | 2.22 | 2.45 |
|  | 9 | **H2N3F2** | 2.88 | 3.73 | 1.90 | 3.88 | 4.32 | 4.65 | 4.26 | 4.51 |
|  | 10 | **H2N4F2** | 2.08 | 2.90 | 2.23 | 2.46 | 3.36 | 3.32 | 4.11 | 3.56 |

**Table G.**

- 1. **Linearity**

| Sample amount (μg) | Glycan composition | MALDI-TOF-MS data of permethylated *O*-glycans released by semi-automated reductive β-elimination | | | | | | | |
| --- | --- | --- | --- | --- | --- | --- | --- | --- | --- |
|  |  | **Replicate A** | | **Replicate B** | | **Replicate C** | | **Data** | |
|  |  | **Intensity** | **RI (%)** | **Intensity** | **RI (%)** | **Intensity** | **RI (%)** | **SD** | **CV** |
| 5 | **N1S1** | 134761 | **31.53** | 165162 | **31.49** | 151348 | **31.46** | 0.04 | **0.12** |
|  | **N1Sg1** | 62618 | **14.65** | 80514 | **15.35** | 74427 | **15.47** | 0.44 | **2.91** |
|  | **N2S1** | 148326 | **34.71** | 179391 | **34.20** | 164684 | **34.23** | 0.28 | **0.83** |
|  | **N2Sg1** | 81676 | **19.11** | 99435 | **18.96** | 90697 | **18.85** | 0.13 | **0.69** |
| 10 | **N1S1** | 284696 | **30.07** | 195283 | **30.27** | 296944 | **34.84** | 2.70 | **8.51** |
|  | **N1Sg1** | 150409 | **15.89** | 97676 | **15.14** | 112550 | **13.21** | 1.38 | **9.38** |
|  | **N2S1** | 322809 | **34.10** | 227736 | **35.30** | 310694 | **36.46** | 1.18 | **3.34** |
|  | **N2Sg1** | 188759 | **19.94** | 124431 | **19.29** | 132022 | **15.49** | 2.40 | **13.17** |
| 50 | **N1S1** | 376050 | **32.33** | 263210 | **32.41** | 255822 | **32.59** | 1.36 | **4.08** |
|  | **N1Sg1** | 176866 | **15.21** | 127211 | **15.66** | 121313 | **15.45** | 1.36 | **9.21** |
|  | **N2S1** | 399618 | **34.36** | 273366 | **33.66** | 265699 | **33.85** | 1.57 | **4.52** |
|  | **N2Sg1** | 210497 | **18.10** | 148442 | **18.28** | 142162 | **18.11** | 1.56 | **9.03** |
| 100 | **N1S1** | 200395 | **32.50** | 276334 | **35.19** | 285352 | **33.41** | 1.37 | **4.06** |
|  | **N1Sg1** | 93728 | **15.20** | 128010 | **16.30** | 132718 | **15.54** | 0.56 | **3.60** |
|  | **N2S1** | 212318 | **34.43** | 248364 | **31.63** | 284795 | **33.34** | 1.41 | **4.27** |
|  | **N2Sg1** | 110158 | **17.87** | 132526 | **16.88** | 151320 | **17.72** | 0.53 | **3.05** |
| 200 | **N1S1** | 3291 | **36.80** | 10232 | **36.78** | 4495 | **37.00** | 0.12 | **0.33** |
|  | **N1Sg1** | 1665 | **18.62** | 4906 | **17.64** | 1851 | **15.24** | 1.74 | **10.14** |
|  | **N2S1** | 2519 | **28.17** | 8137 | **29.25** | 3774 | **31.06** | 1.46 | **4.96** |
|  | **N2Sg1** | 1468 | **16.42** | 4544 | **16.33** | 2029 | **16.70** | 0.19 | **1.17** |

**Table H.**

| Peak no. | Sample amount (μg) | Glycan composition | MALDI-TOF-MS data of permethylated *O*-glycans released by semi-automated reductive β-elimination | | | | | | |
| --- | --- | --- | --- | --- | --- | --- | --- | --- | --- |
|  |  |  | **Replicate A** | | **Replicate B** | | **Replicate C** | | **Avg. RI (%)** |
|  |  |  | **Intensity** | **RI (%)** | **Intensity** | **RI (%)** | **Intensity** | **RI (%)** |  |
| 1 | 50 | **N1S1** | 376050 | **27.53** | 263210 | **27.68** | 255822 | **27.94** | **27.72** |
| 2 |  | **N1Sg1** | 176866 | **12.95** | 127211 | **13.38** | 121313 | **13.25** | **13.19** |
| 3 |  | **N2S1** | 399618 | **29.25** | 273366 | **28.75** | 265699 | **29.02** | **29.01** |
| 4 |  | **H1N2F1** | 56958 | **4.17** | 35963 | **3.78** | 37575 | **4.10** | **4.02** |
| 5 |  | **N2Sg1** | 210497 | **15.41** | 148442 | **15.61** | 142162 | **15.53** | **15.52** |
| 6 |  | **H1N3F1** | 35345 | **2.59** | 21584 | **2.27** | 21294 | **2.33** | **2.39** |
| 7 |  | **H1N2F1S1** | 44840 | **3.28** | 31037 | **3.26** | 27790 | **3.04** | **3.19** |
| 8 |  | **H1N3F1S1** | 17938 | **1.31** | 13539 | **1.42** | 12680 | **1.38** | **1.37** |
| 9 |  | **H2N3F2** | 27728 | **2.03** | 21010 | **2.21** | 17258 | **1.88** | **2.04** |
| 10 |  | **H2N4F2** | 20197 | **1.48** | 15391 | **1.62** | 14042 | **1.53** | **1.54** |
| 1 | 100 | **N1S1** | 200395 | **27.72** | 276334 | **30.39** | 285352 | **28.55** | **28.89** |
| 2 |  | **N1Sg1** | 93728 | **12.97** | 128010 | **14.08** | 132718 | **13.28** | **13.44** |
| 3 |  | **N2S1** | 212318 | **29.37** | 248364 | **27.31** | 284795 | **28.49** | **28.39** |
| 4 |  | **H1N2F1** | 33724 | **4.66** | 35451 | **3.90** | 40359 | **4.04** | **4.20** |
| 5 |  | **N2Sg1** | 110158 | **15.24** | 132526 | **14.57** | 151320 | **15.14** | **14.98** |
| 6 |  | **H1N3F1** | 15027 | **2.08** | 17306 | **1.90** | 20130 | **2.01** | **2.00** |
| 7 |  | **H1N2F1S1** | 22436 | **3.10** | 27637 | **3.04** | 33104 | **3.31** | **3.15** |
| 8 |  | **H1N3F1S1** | 9438 | **1.31** | 12434 | **1.37** | 15064 | **1.51** | **1.39** |
| 9 |  | **H2N3F2** | 14343 | **1.98** | 17961 | **1.98** | 21087 | **2.11** | **2.02** |
| 10 |  | **H2N4F2** | 11363 | **1.57** | 13247 | **1.46** | 15666 | **1.57** | **1.53** |
| 1 | 200 | **N1S1** | 3291 | **32.04** | 10232 | **31.90** | 4495 | **31.28** | **31.74** |
| 2 |  | **N1Sg1** | 1665 | **16.21** | 4906 | **15.30** | 1851 | **12.88** | **14.80** |
| 3 |  | **N2S1** | 2519 | **24.52** | 8137 | **25.37** | 3774 | **26.27** | **25.39** |
| 4 |  | **H1N2F1** | 460 | **4.48** | 1446 | **4.51** | 652 | **4.54** | **4.51** |
| 5 |  | **N2Sg1** | 1468 | **14.29** | 4544 | **14.17** | 2029 | **14.12** | **14.19** |
| 6 |  | **H1N3F1** | 205 | **2.00** | 516 | **1.61** | 287 | **2.00** | **1.87** |
| 7 |  | **H1N2F1S1** | 273 | **2.66** | 910 | **2.84** | 487 | **3.39** | **2.96** |
| 8 |  | **H1N3F1S1** | 99.6 | **0.97** | 374 | **1.17** | 257 | **1.79** | **1.31** |
| 9 |  | **H2N3F2** | 196 | **1.91** | 639 | **1.99** | 308 | **2.14** | **2.01** |
| 10 |  | **H2N4F2** | 95.7 | **0.93** | 368 | **1.15** | 228 | **1.59** | **1.22** |

**Table I.**


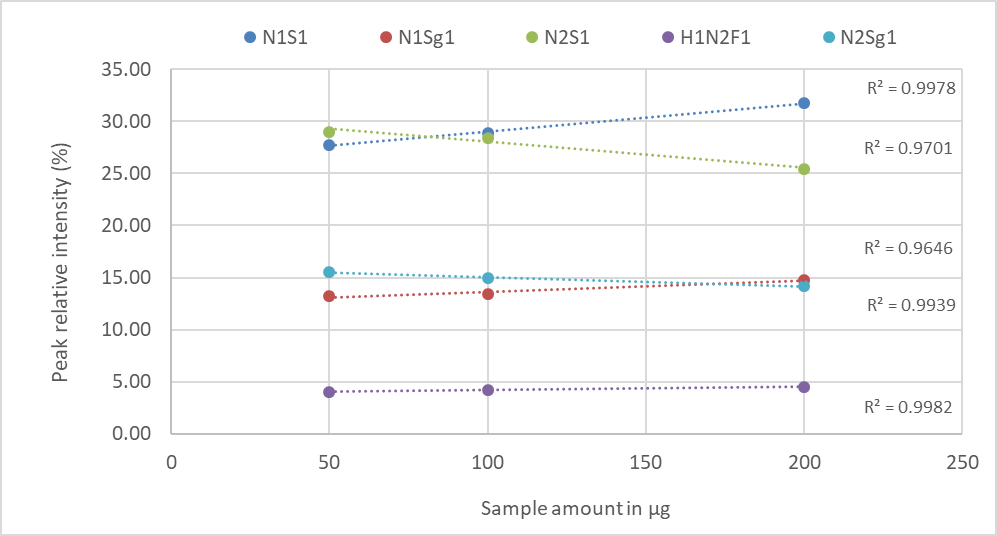

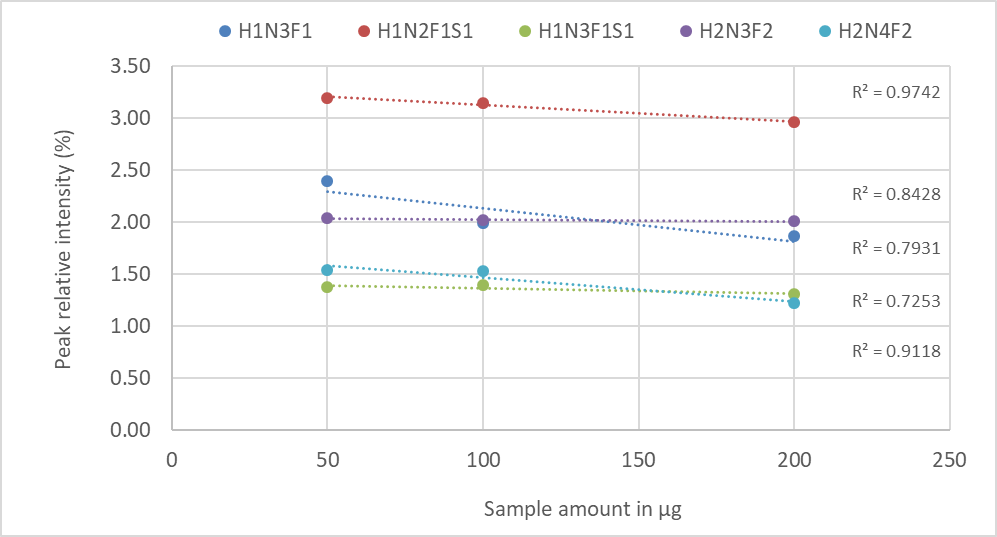


**Figure C.**

- 1. **Limit of Quantitation and Limit of Detection**

| S/N ratios from MALDI-TOF-MS data of permethylated BSM type I-S *O*-glycans released by semi-automated reductive β-elimination | | | | | | | | | | | | | | | | |
| --- | --- | --- | --- | --- | --- | --- | --- | --- | --- | --- | --- | --- | --- | --- | --- | --- |
| Peak no. | **Glycan composition** | **RA (%)** | **5 μg** | **10 μg** | **50 μg** | **100 μg** | **200 μg** |  | **Peak no.** | **Glycan composition** | **RA (%)** | **5 μg** | **10 μg** | **50 μg** | **100 μg** | **200 μg** |
| 1 | **N2** | **0.84** | 10 | 11 | 17 | 11 | 9 |  | 21 | **H1N2F2** | **0.24** | 5 | 6 | 5 | 3 | 2 |
| 2 | **N1S1** | **15.87** | 153 | 224 | 228 | 256 | 207 |  | 22 | **H1N1S2** | **0.66** | 11 | 16 | 12 | 13 | 7 |
| 3 | **H1N1F1** | **1.55** | 15 | 19 | 26 | 22 | 22 |  | 23 | **H1N1S1Sg1** | **0.30** | 5 | 7 | 4 | 4 | 4 |
| 4 | **N1Sg1** | **7.38** | 82 | 86 | 105 | 115 | 97 |  | 24 | **H1N2F1S1** | **3.51** | 68 | 73 | 81 | 59 | 33 |
| 5 | **H1N2** | **0.54** | 6 | 5 | 7 | 5 | 4 |  | 25 | **H2N2F2** | **3.68** | 82 | 7 | 110 | 68 | 48 |
| 6 | **N3** | **0.76** | 9 | 6 | 9 | 6 | 6 |  | 26 | **H2N2S1** | **2.06** | 41 | 37 | 46 | 33 | 21 |
| 7 | **H1N1S1** | **1.23** | 15 | 12 | 16 | 14 | 14 |  | 27 | **H3N2F1** | **0.43** | 7 | 9 | 8 | 4 | 4 |
| 8 | **H1N1Sg1** | **0.64** | 9 | 6 | 9 | 6 | 8 |  | 28 | **H1N3F2** | **0.66** | 12 | 10 | 16 | 9 | 7 |
| 9 | **N2S1** | **20.89** | 274 | 244 | 308 | 232 | 187 |  | 29 | **H2N3F1** | **0.51** | 10 | 12 | 12 | 7 | 2 |
| 10 | **H1N2F1** | **3.40** | 43 | 35 | 59 | 33 | 33 |  | 30 | **H1N4F1** | **0.96** | 18 | 17 | 22 | 14 | 9 |
| 11 | **N2Sg1** | **11.01** | 156 | 104 | 162 | 124 | 105 |  | 31 | **H1N2F2S1** | **1.80** | 31 | 33 | 42 | 24 | 15 |
| 12 | **H2N2** | **0.52** | 7 | 5 | 8 | 5 | 6 |  | 32 | **H2N2F3** | **1.21** | 22 | 21 | 27 | 14 | 13 |
| 13 | **H1N3** | **0.43** | 6 | 4 | 6 | 4 | 4 |  | 33 | **H1N2F2Sg1** | **1.24** | 21 | 20 | 28 | 17 | 10 |
| 14 | **H1N1F1S1** | **0.87** | 10 | 9 | 11 | 8 | 7 |  | 34 | **H2N2F1Sg1** | **0.70** | 13 | 11 | 11 | 7 | 9 |
| 15 | **H1N1F1Sg1** | **0.39** | 5 | 4 | 5 | 4 | 2 |  | 35 | **H1N3F1S1** | **1.92** | 33 | 40 | 34 | 25 | 15 |
| 16 | **H1N2F2** | **0.28** | 3 | 2 | 5 | 2 | 2 |  | 36 | **H2N3F2** | **3.00** | 51 | 57 | 60 | 35 | 25 |
| 17 | **H1N2S1** | **1.23** | 14 | 11 | 14 | 11 | 11 |  | 37 | **H1N3F1Sg1** | **1.18** | 22 | 21 | 22 | 15 | 9 |
| 18 | **H2N2F1** | **1.05** | 12 | 9 | 15 | 9 | 8 |  | 38 | **H2N3F3** | **0.80** | 13 | 13 | 14 | 8 | 8 |
| 19 | **H1N2Sg1** | **0.76** | 12 | 11 | 12 | 11 | 6 |  | 39 | **H3N3S1** | **0.86** | 17 | 12 | 15 | 9 | 8 |
| 20 | **H1N3F1** | **2.08** | 31 | 33 | 38 | 27 | 16 |  | 40 | **H2N4F2** | **2.58** | 41 | 52 | 51 | 27 | 18 |

**Table J.**

- 1. **Specificity**

(plate position **F5**)

(plate position **H1**)

**Figure D.**

- 1. **Cross-over contamination**

**Figure E.**

50 μg BSM

(plate position **H1**)

0.0

0.5

1.0

1.5

2.0

5

x10

0.0

0.5

1.0

1.5

2.0

5

x10

0.0

0.5

1.0

1.5

2.0

5

x10

0.0

0.5

1.0

1.5

2.0

5

x10

0.0

0.5

1.0

1.5

2.0

5

x10

0.0

0.5

1.0

1.5

2.0

5

x10

600

800

1000

1200

1400

1600

1800

m/z

Water blank

(plate position **G1**)

Water blank

(plate position **A3**)

Signal intensity

575.32

691.36

708.38

721.37

779.41

820.44

895.46

925.47

936.49

953.50

966.49

983.51

1024.54

1069.55

1099.56

1127.59

1140.59

1198.63

1131.69

1402.73

1821.95

Water blank

(plate position **E4**)

**Additional experimental data – Section 4**

Water blank

(plate position **E6**)

Water blank

(plate position **F5**)

- 1. **Comparison of the different incubation methods**

**Figure F.**

- 1. **MS/MS fragmentation of O-linked glycans from BSM type I-S**

**
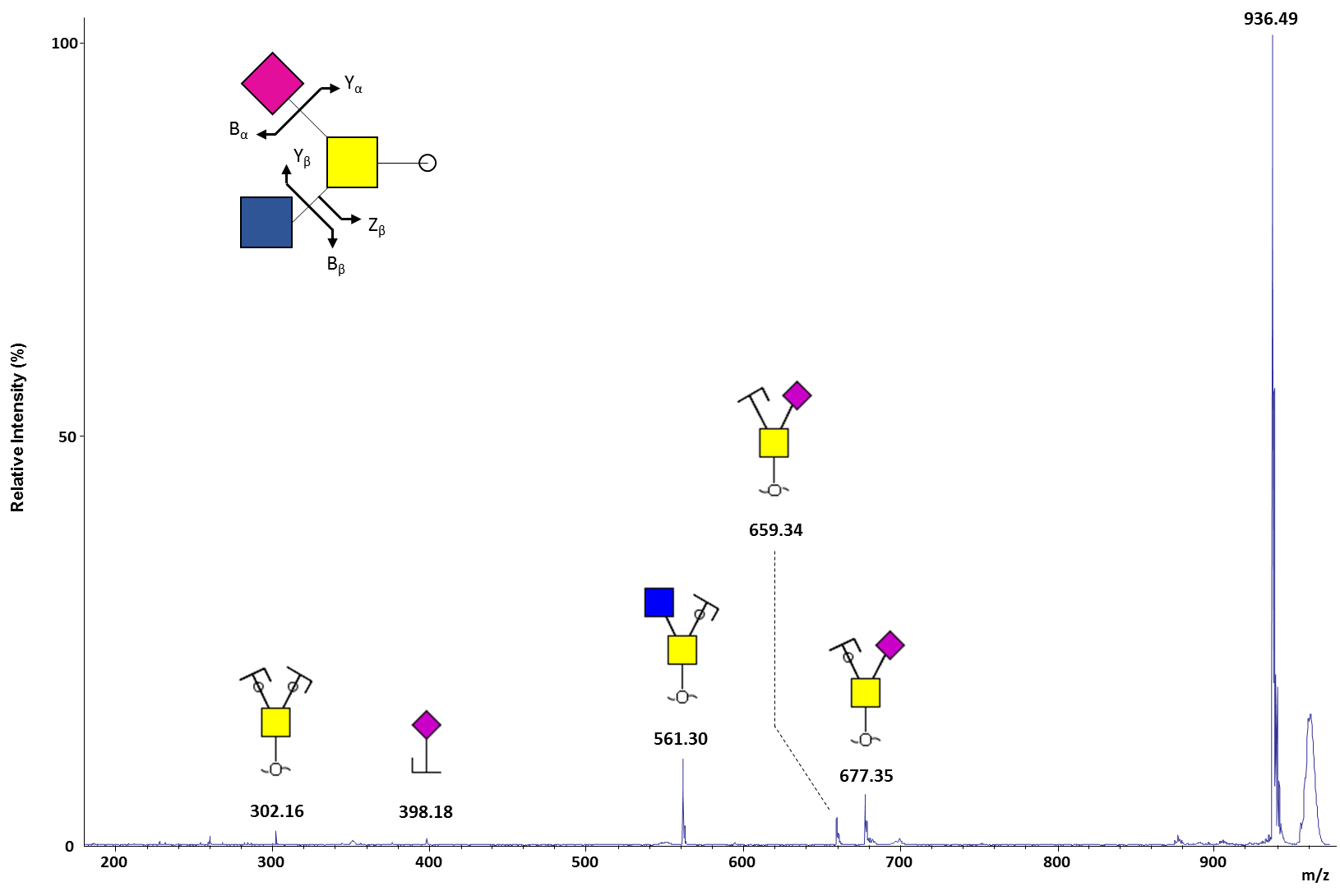
**

**Figure G.**

**
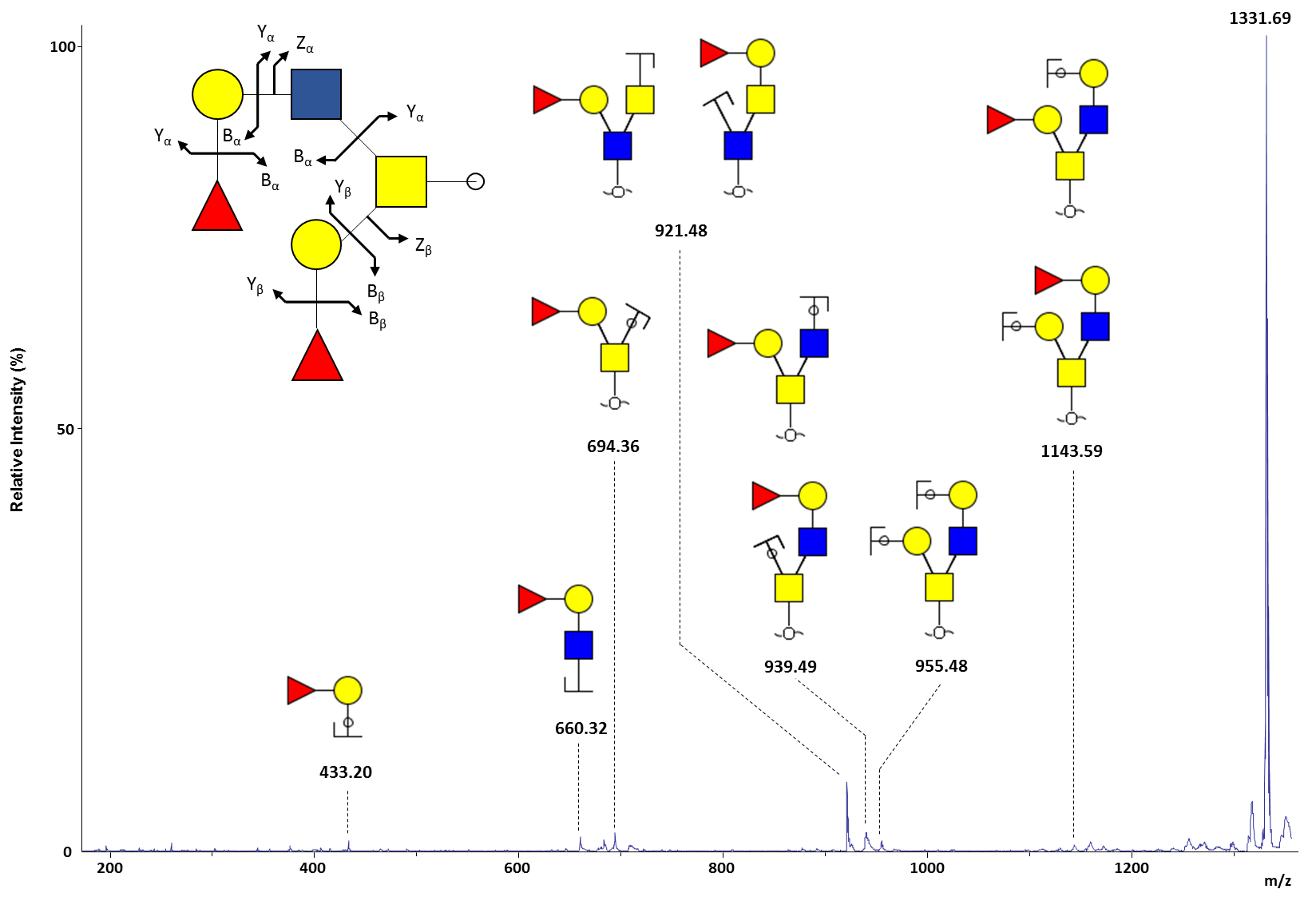
**

**Figure H.**

**
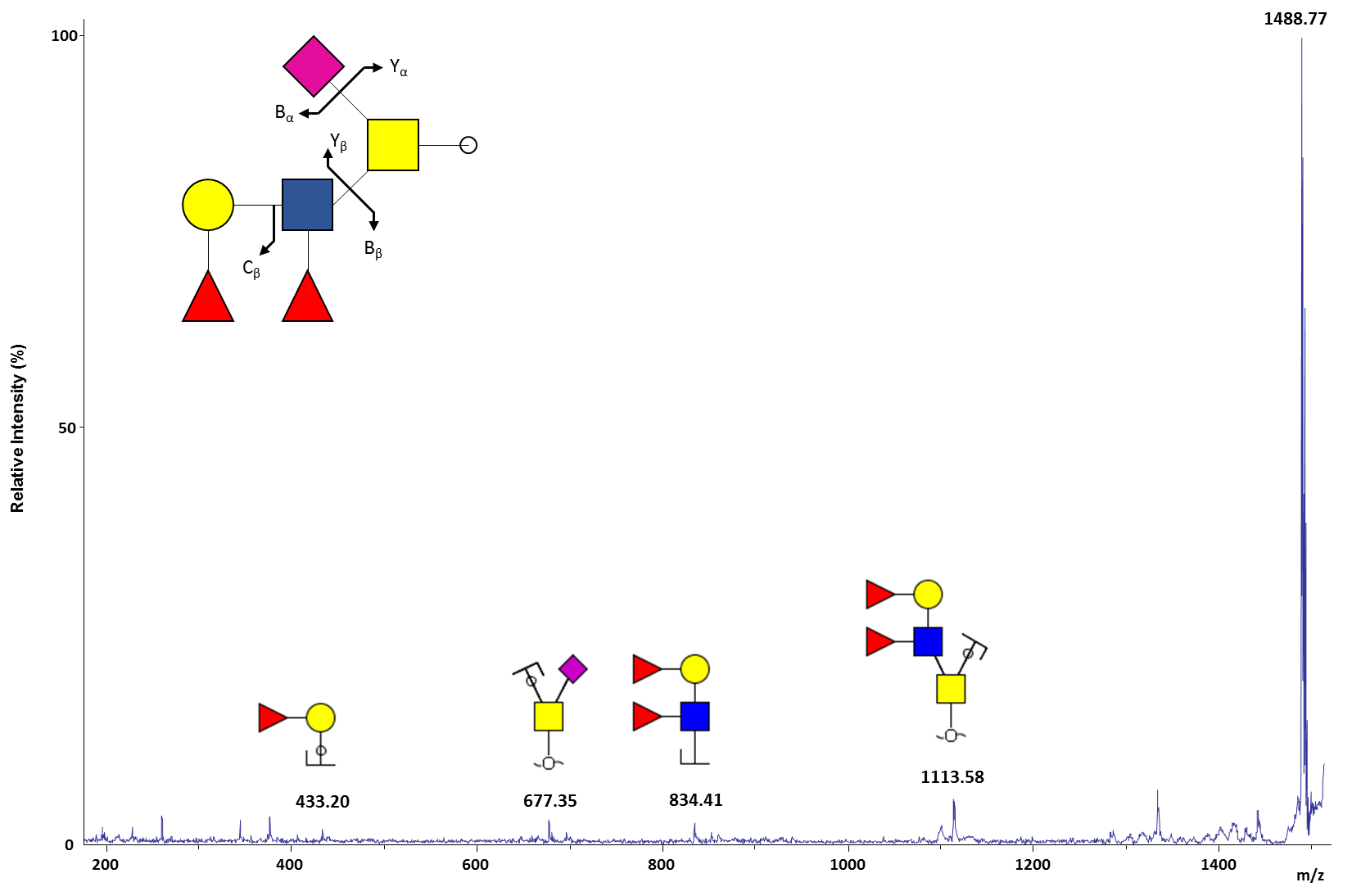
**

**Figure I.**

**Additional data** **– Section 5**

(data points behind all statistical studies)

1. **Accuracy (ten representative *O*-glycans)**

| MALDI-TOF-MS data of permethylated *O*-glycans released by manual in-solution reductive β-elimination | | | | | | | | | | |
| --- | --- | --- | --- | --- | --- | --- | --- | --- | --- | --- |
| Peak no. | **Glycan composition** | **Replicate A** | | **Replicate B** | | **Replicate C** | | **Data** | | |
|  |  | **Area** | **RA (%)** | **Area** | **RA (%)** | **Area** | **RA (%)** | **Avg. RA (%)** | **SD** | **CV** |
| 1 | **N1S1** | 83177 | 22.67 | 39933 | 23.63 | 128937 | 23.49 | **23.26** | 0.52 | **2.22** |
| 2 | **N1Sg1** | 39813 | 10.85 | 16757 | 9.92 | 65949 | 12.02 | **10.93** | 1.05 | **9.63** |
| 3 | **N2S1** | 101310 | 27.62 | 50047 | 29.61 | 154577 | 28.16 | **28.46** | 1.03 | **3.63** |
| 4 | **H1N2F1** | 15920 | 4.34 | 6878 | 4.07 | 23283 | 4.24 | **4.22** | 0.14 | **3.24** |
| 5 | **N2Sg1** | 56620 | 15.43 | 23904 | 14.14 | 89916 | 16.38 | **15.32** | 1.12 | **7.33** |
| 6 | **H1N3F1** | 10066 | 2.74 | 4087 | 2.42 | 12664 | 2.31 | **2.49** | 0.23 | **9.11** |
| 7 | **H1N2F1S1** | 16076 | 4.38 | 7456 | 4.41 | 24595 | 4.48 | **4.42** | 0.05 | **1.15** |
| 8 | **H1N3F1S1** | 9871 | 2.69 | 4910 | 2.91 | 12189 | 2.22 | **2.61** | 0.35 | **13.44** |
| 9 | **H2N3F2** | 17025 | 4.64 | 7120 | 4.21 | 19266 | 3.51 | **4.12** | 0.57 | **13.85** |
| 10 | **H2N4F2** | 16987 | 4.63 | 7903 | 4.68 | 17498 | 3.19 | **4.17** | 0.85 | **20.32** |

| MALDI-TOF-MS data of permethylated *O*-glycans released by semi-automated reductive β-elimination | | | | | | | | | | |
| --- | --- | --- | --- | --- | --- | --- | --- | --- | --- | --- |
| Peak no. | **Glycan composition** | **Replicate A** | | **Replicate B** | | **Replicate C** | | **Data** | | |
|  |  | **Area** | **RA (%)** | **Area** | **RA (%)** | **Area** | **RA (%)** | **Avg. RA (%)** | **SD** | **CV** |
| 1 | **N1S1** | 57169 | 20.64 | 55655 | 20.95 | 84003 | 20.70 | **20.76** | 0.16 | **0.79** |
| 2 | **N1Sg1** | 28371 | 10.24 | 26913 | 10.13 | 40007 | 9.86 | **10.08** | 0.20 | **1.96** |
| 3 | **N2S1** | 79290 | 28.62 | 77158 | 29.04 | 118521 | 29.20 | **28.95** | 0.30 | **1.03** |
| 4 | **H1N2F1** | 13533 | 4.89 | 14110 | 5.31 | 22942 | 5.65 | **5.28** | 0.38 | **7.27** |
| 5 | **N2Sg1** | 44768 | 16.16 | 42750 | 16.09 | 63722 | 15.70 | **15.98** | 0.25 | **1.55** |
| 6 | **H1N3F1** | 8413 | 3.04 | 8302 | 3.12 | 13909 | 3.43 | **3.20** | 0.20 | **6.40** |
| 7 | **H1N2F1S1** | 14259 | 5.15 | 12801 | 4.82 | 21029 | 5.18 | **5.05** | 0.20 | **3.97** |
| 8 | **H1N3F1S1** | 7726 | 2.79 | 6636 | 2.50 | 9764 | 2.41 | **2.57** | 0.20 | **7.81** |
| 9 | **H2N3F2** | 12374 | 4.47 | 11210 | 4.22 | 17439 | 4.30 | **4.33** | 0.13 | **2.93** |
| 10 | **H2N4F2** | 11110 | 4.01 | 10156 | 3.82 | 14540 | 3.58 | **3.80** | 0.21 | **5.64** |

**Table K.**

1. **Accuracy (detected *O*-glycans)**

| MALDI-TOF-MS data of permethylated *O*-glycans released by manual in-solution reductive β-elimination | | | | | | | | | | |
| --- | --- | --- | --- | --- | --- | --- | --- | --- | --- | --- |
| Peak no. | **Glycan composition** | **Replicate A** | | **Replicate B** | | **Replicate C** | | **Data** | | |
|  |  | **Area** | **RA (%)** | **Area** | **RA (%)** | **Area** | **RA (%)** | **Avg. RA (%)** | **SD** | **CV** |
| 1 | **N2** | 3357 | 0.66 | 1043 | 0.46 | 3395 | 0.46 | **0.52** | 0.12 | **22.01** |
| 2 | **N1S1** | 83177 | 16.31 | 39933 | 17.54 | 128937 | 17.40 | **17.08** | 0.67 | **3.94** |
| 3 | **H1N1F1** | 7915 | 1.55 | 3029 | 1.33 | 10886 | 1.47 | **1.45** | 0.11 | **7.72** |
| 4 | **N1Sg1** | 39813 | 7.81 | 16757 | 7.36 | 65949 | 8.90 | **8.02** | 0.79 | **9.87** |
| 5 | **H1N2** | 2750 | 0.54 | 889 | 0.39 | 3045 | 0.41 | **0.45** | 0.08 | **18.04** |
| 6 | **N3** | 4100 | 0.80 | 1337 | 0.59 | 4301 | 0.58 | **0.66** | 0.13 | **19.34** |
| 7 | **H1N1S1** | 5566 | 1.09 | 3019 | 1.33 | 9701 | 1.31 | **1.24** | 0.13 | **10.53** |
| 8 | **H1N1Sg1** | 2742 | 0.54 | 1117 | 0.49 | 4360 | 0.59 | **0.54** | 0.05 | **9.07** |
| 9 | **N2S1** | 101310 | 19.87 | 50047 | 21.98 | 154577 | 20.86 | **20.90** | 1.06 | **5.07** |
| 10 | **H1N2F1** | 15920 | 3.12 | 6878 | 3.02 | 23283 | 3.14 | **3.09** | 0.06 | **2.09** |
| 11 | **N2Sg1** | 56620 | 11.10 | 23904 | 10.50 | 89916 | 12.13 | **11.25** | 0.83 | **7.35** |
| 12 | **H2N2** | 2416 | 0.47 | 974 | 0.43 | 2802 | 0.38 | **0.43** | 0.05 | **11.21** |
| 13 | **H1N3** | 1917 | 0.38 | 726 | 0.32 | 2567 | 0.35 | **0.35** | 0.03 | **8.22** |
| 14 | **H1N1F1S1** | 3757 | 0.74 | 2333 | 1.02 | 7388 | 1.00 | **0.92** | 0.16 | **17.28** |
| 15 | **H1N1F1Sg1** | 1996 | 0.39 | 1061 | 0.47 | 3684 | 0.50 | **0.45** | 0.05 | **12.04** |
| 16 | **H1N2F2** | 1369 | 0.27 | 570 | 0.25 | 1826 | 0.25 | **0.26** | 0.01 | **4.60** |
| 17 | **H1N2S1** | 5281 | 1.04 | 2675 | 1.17 | 8306 | 1.12 | **1.11** | 0.07 | **6.33** |
| 18 | **H2N2F1** | 4772 | 0.94 | 1983 | 0.87 | 6441 | 0.87 | **0.89** | 0.04 | **4.25** |
| 19 | **H1N2Sg1** | 3589 | 0.70 | 1451 | 0.64 | 5114 | 0.69 | **0.68** | 0.04 | **5.18** |
| 20 | **H1N3F1** | 10066 | 1.97 | 4087 | 1.80 | 12664 | 1.71 | **1.83** | 0.14 | **7.40** |
| 21 | **H1N2F2** | 1249 | 0.24 | 464 | 0.20 | 1173 | 0.16 | **0.20** | 0.04 | **21.41** |
| 22 | **H1N1S2** | 3748 | 0.73 | 2045 | 0.90 | 5903 | 0.80 | **0.81** | 0.08 | **10.18** |
| 23 | **H1N1S1Sg1** | 1208 | 0.24 | 697 | 0.31 | 1875 | 0.25 | **0.27** | 0.04 | **13.66** |
| 24 | **H1N2F1S1** | 16076 | 3.15 | 7456 | 3.27 | 24595 | 3.32 | **3.25** | 0.09 | **2.66** |
| 25 | **H2N2F2** | 20601 | 4.04 | 8601 | 3.78 | 30137 | 4.07 | **3.96** | 0.16 | **4.03** |
| 26 | **H2N2S1** | 9738 | 1.91 | 4051 | 1.78 | 14689 | 1.98 | **1.89** | 0.10 | **5.44** |
| 27 | **H3N2F1** | 2312 | 0.45 | 797 | 0.35 | 1927 | 0.26 | **0.35** | 0.10 | **18.29** |
| 28 | **H1N3F2** | 3123 | 0.61 | 1043 | 0.46 | 3590 | 0.48 | **0.52** | 0.08 | **15.92** |
| 29 | **H2N3F1** | 3066 | 0.60 | 1166 | 0.51 | 3189 | 0.43 | **0.51** | 0.09 | **16.61** |
| 30 | **H1N4F1** | 5862 | 1.15 | 1784 | 0.78 | 5425 | 0.73 | **0.89** | 0.23 | **25.62** |
| 31 | **H1N2F2S1** | 8329 | 1.63 | 3729 | 1.64 | 12291 | 1.66 | **1.64** | 0.01 | **0.82** |
| 32 | **H2N2F3** | 5640 | 1.11 | 2338 | 1.03 | 7831 | 1.06 | **1.06** | 0.04 | **3.75** |
| 33 | **H1N2F2Sg1** | 6330 | 1.24 | 2440 | 1.07 | 8721 | 1.18 | **1.16** | 0.09 | **7.36** |
| 34 | **H2N2F1Sg1** | 4091 | 0.80 | 1222 | 0.54 | 3764 | 0.51 | **0.62** | 0.16 | **26.35** |
| 35 | **H1N3F1S1** | 9871 | 1.94 | 4910 | 2.16 | 12189 | 1.64 | **1.91** | 0.26 | **13.42** |
| 36 | **H2N3F2** | 17025 | 3.34 | 7120 | 3.13 | 19266 | 2.60 | **3.02** | 0.38 | **12.59** |
| 37 | **H1N3F1Sg1** | 6541 | 1.28 | 2804 | 1.23 | 7695 | 1.04 | **1.18** | 0.13 | **10.88** |
| 38 | **H2N3F3** | 4213 | 0.83 | 1759 | 0.77 | 4949 | 0.67 | **0.76** | 0.08 | **10.65** |
| 39 | **H3N3S1** | 5546 | 1.09 | 1532 | 0.67 | 5188 | 0.70 | **0.82** | 0.23 | **28.28** |
| 40 | **H2N4F2** | 16987 | 3.33 | 7903 | 3.47 | 17498 | 2.36 | **3.05** | 0.60 | **19.79** |

| MALDI-TOF-MS data of permethylated *O*-glycans released by semi-automated reductive β-elimination | | | | | | | | | | |
| --- | --- | --- | --- | --- | --- | --- | --- | --- | --- | --- |
| Peak no. | **Glycan composition** | **Replicate A** | | **Replicate B** | | **Replicate C** | | **Data** | | |
|  |  | **Area** | **RA (%)** | **Area** | **RA (%)** | **Area** | **RA (%)** | **Avg. RA (%)** | **SD** | **CV** |
| 1 | **N2** | 2572 | 0.66 | 3188 | 0.85 | 5371 | 0.93 | **0.81** | 0.14 | **17.33** |
| 2 | **N1S1** | 57169 | 14.58 | 55655 | 14.82 | 84003 | 14.54 | **14.65** | 1.15 | **1.02** |
| 3 | **H1N1F1** | 5894 | 1.50 | 6285 | 1.67 | 9774 | 1.69 | **1.62** | 0.10 | **6.41** |
| 4 | **N1Sg1** | 28371 | 7.24 | 26913 | 7.17 | 40007 | 6.93 | **7.11** | 0.16 | **2.29** |
| 5 | **H1N2** | 2057 | 0.52 | 2229 | 0.59 | 3460 | 0.60 | **0.57** | 0.04 | **7.23** |
| 6 | **N3** | 2779 | 0.71 | 3206 | 0.85 | 4783 | 0.83 | **0.80** | 0.08 | **9.70** |
| 7 | **H1N1S1** | 4542 | 1.16 | 4386 | 1.17 | 6525 | 1.13 | **1.15** | 0.02 | **1.73** |
| 8 | **H1N1Sg1** | 2438 | 0.62 | 2291 | 0.61 | 3545 | 0.61 | **0.62** | 0.01 | **0.99** |
| 9 | **N2S1** | 79290 | 20.23 | 77158 | 20.54 | 118521 | 20.52 | **20.43** | 0.18 | **0.87** |
| 10 | **H1N2F1** | 13533 | 3.45 | 14110 | 3.76 | 22942 | 3.97 | **3.73** | 0.26 | **7.01** |
| 11 | **N2Sg1** | 44768 | 11.42 | 42750 | 11.38 | 63722 | 11.03 | **11.28** | 0.21 | **1.89** |
| 12 | **H2N2** | 1972 | 0.50 | 2197 | 0.58 | 3000 | 0.52 | **0.54** | 0.04 | **8.09** |
| 13 | **H1N3** | 1684 | 0.43 | 1871 | 0.50 | 2424 | 0.42 | **0.45** | 0.04 | **9.52** |
| 14 | **H1N1F1S1** | 3397 | 0.87 | 3088 | 0.82 | 4922 | 0.85 | **0.85** | 0.02 | **2.67** |
| 15 | **H1N1F1Sg1** | 1744 | 0.44 | 1534 | 0.41 | 2444 | 0.42 | **0.43** | 0.02 | **4.31** |
| 16 | **H1N2F2** | 1187 | 0.30 | 1437 | 0.38 | 2472 | 0.43 | **0.37** | 0.06 | **17.08** |
| 17 | **H1N2S1** | 4697 | 1.20 | 4358 | 1.16 | 6590 | 1.14 | **1.17** | 0.03 | **2.49** |
| 18 | **H2N2F1** | 4271 | 1.09 | 3999 | 1.06 | 6571 | 1.14 | **1.10** | 0.04 | **3.38** |
| 19 | **H1N2Sg1** | 3077 | 0.78 | 2858 | 0.76 | 4490 | 0.78 | **0.77** | 0.01 | **1.58** |
| 20 | **H1N3F1** | 8413 | 2.15 | 8302 | 2.21 | 13909 | 2.41 | **2.25** | 0.14 | **6.06** |
| 21 | **H1N2F2** | 870 | 0.22 | 937 | 0.25 | 1332 | 0.23 | **0.23** | 0.01 | **6.02** |
| 22 | **H1N1S2** | 2483 | 0.63 | 2023 | 0.54 | 3123 | 0.54 | **0.57** | 0.05 | **9.48** |
| 23 | **H1N1S1Sg1** | 864 | 0.22 | 814 | 0.22 | 1066 | 0.18 | **0.21** | 0.02 | **9.51** |
| 24 | **H1N2F1S1** | 14259 | 3.64 | 12801 | 3.41 | 21029 | 3.64 | **3.56** | 0.13 | **3.74** |
| 25 | **H2N2F2** | 18404 | 4.69 | 16907 | 4.50 | 28515 | 4.94 | **4.71** | 0.22 | **4.63** |
| 26 | **H2N2S1** | 8334 | 2.13 | 7596 | 2.02 | 12079 | 2.09 | **2.08** | 0.05 | **2.53** |
| 27 | **H3N2F1** | 1340 | 0.34 | 1385 | 0.37 | 2116 | 0.37 | **0.36** | 0.01 | **4.15** |
| 28 | **H1N3F2** | 2602 | 0.66 | 2675 | 0.71 | 4354 | 0.75 | **0.71** | 0.05 | **6.35** |
| 29 | **H2N3F1** | 2415 | 0.62 | 2174 | 0.58 | 3144 | 0.54 | **0.58** | 0.04 | **6.18** |
| 30 | **H1N4F1** | 3997 | 1.02 | 3932 | 1.05 | 5835 | 1.01 | **1.03** | 0.02 | **1.86** |
| 31 | **H1N2F2S1** | 7629 | 1.95 | 7048 | 1.88 | 11406 | 1.97 | **1.93** | 0.05 | **2.61** |
| 32 | **H2N2F3** | 4936 | 1.26 | 4401 | 1.17 | 7252 | 1.26 | **1.23** | 0.05 | **4.02** |
| 33 | **H1N2F2Sg1** | 5262 | 1.34 | 4835 | 1.29 | 7765 | 1.34 | **1.32** | 0.03 | **2.44** |
| 34 | **H2N2F1Sg1** | 2427 | 0.62 | 2312 | 0.62 | 3219 | 0.56 | **0.60** | 0.03 | **5.81** |
| 35 | **H1N3F1S1** | 7726 | 1.97 | 6636 | 1.77 | 9764 | 1.69 | **1.81** | 0.14 | **8.01** |
| 36 | **H2N3F2** | 12374 | 3.16 | 11210 | 2.98 | 17439 | 3.02 | **3.05** | 0.09 | **2.97** |
| 37 | **H1N3F1Sg1** | 4888 | 1.25 | 4297 | 1.14 | 5989 | 1.04 | **1.14** | 0.10 | **9.19** |
| 38 | **H2N3F3** | 3063 | 0.78 | 2668 | 0.71 | 4019 | 0.70 | **0.73** | 0.05 | **6.27** |
| 39 | **H3N3S1** | 3174 | 0.81 | 2937 | 0.78 | 4105 | 0.71 | **0.77** | 0.05 | **6.65** |
| 40 | **H2N4F2** | 11110 | 2.83 | 10156 | 2.70 | 14540 | 2.52 | **2.69** | 0.16 | **5.93** |

**Table L.**

1. **Repeatability (ten representative *O*-glycans)**

| MALDI-TOF-MS data of permethylated *O*-glycans released by semi-automated reductive β-elimination | | | | | | | | | | | | | | | | | | | | |
| --- | --- | --- | --- | --- | --- | --- | --- | --- | --- | --- | --- | --- | --- | --- | --- | --- | --- | --- | --- | --- |
| Peak no. | **Glycan composition** | **Replicate A** | | **Replicate B** | | **Replicate C** | | **Replicate D** | | **Replicate E** | | **Replicate F** | | **Replicate G** | | **Replicate H** | | **Data** | | |
|  |  | **Area** | **RA (%)** | **Area** | **RA (%)** | **Area** | **RA (%)** | **Area** | **RA (%)** | **Area** | **RA (%)** | **Area** | **RA (%)** | **Area** | **RA (%)** | **Area** | **RA (%)** | **Avg. RA (%)** | **SD** | **CV** |
| 1 | **N1S1** | 67603 | 22.43 | 36740 | 22.80 | 66979 | 23.08 | 102330 | 22.08 | 49883 | 24.14 | 64788 | 23.91 | 66971 | 22.66 | 81042 | 23.01 | **23.02** | 0.70 | **3.06** |
| 2 | **N1Sg1** | 35110 | 11.65 | 19332 | 12.00 | 28342 | 9.77 | 51355 | 11.08 | 24775 | 11.99 | 32112 | 11.85 | 32496 | 11.00 | 40099 | 11.38 | **11.34** | 0.74 | **6.56** |
| 3 | **N2S1** | 83444 | 27.69 | 46125 | 28.62 | 86691 | 29.88 | 129686 | 27.98 | 58101 | 28.12 | 72354 | 26.70 | 82022 | 27.76 | 102911 | 29.22 | **28.25** | 0.98 | **3.48** |
| 4 | **H1N2F1** | 13959 | 4.63 | 6571 | 4.08 | 12153 | 4.19 | 19938 | 4.30 | 8644 | 4.18 | 12179 | 4.49 | 14037 | 4.75 | 15702 | 4.46 | **4.39** | 0.24 | **5.40** |
| 5 | **N2Sg1** | 48297 | 16.02 | 26433 | 16.40 | 40631 | 14.00 | 74549 | 16.09 | 32654 | 15.81 | 41330 | 15.25 | 45716 | 15.47 | 58659 | 16.65 | **15.71** | 0.83 | **5.27** |
| 6 | **H1N3F1** | 8174 | 2.71 | 3728 | 2.31 | 7516 | 2.59 | 13008 | 2.81 | 5155 | 2.50 | 7780 | 2.87 | 8394 | 2.84 | 8709 | 2.47 | **2.64** | 0.20 | **7.65** |
| 7 | **H1N2F1S1** | 13362 | 4.43 | 6646 | 4.12 | 13489 | 4.65 | 23739 | 5.12 | 8390 | 4.06 | 12012 | 4.43 | 13915 | 4.71 | 14250 | 4.05 | **4.45** | 0.37 | **8.42** |
| 8 | **H1N3F1S1** | 7383 | 2.45 | 4025 | 2.50 | 8586 | 2.96 | 12161 | 2.62 | 4750 | 2.30 | 6693 | 2.47 | 7463 | 2.53 | 7494 | 2.13 | **2.49** | 0.24 | **9.69** |
| 9 | **H2N3F2** | 12254 | 4.07 | 5739 | 3.56 | 12668 | 4.37 | 19900 | 4.29 | 7481 | 3.62 | 11707 | 4.32 | 12754 | 4.32 | 12105 | 3.44 | **4.00** | 0.39 | **9.83** |
| 10 | **H2N4F2** | 11807 | 3.92 | 5803 | 3.60 | 13098 | 4.51 | 16766 | 3.62 | 6772 | 3.28 | 10002 | 3.69 | 11715 | 3.96 | 11273 | 3.20 | **3.72** | 0.42 | **11.22** |

**Table M.**

1. **Repeatability (detected *O*-glycans)**

| MALDI-TOF-MS data of permethylated *O*-glycans released by semi-automated reductive β-elimination | | | | | | | | | | | | | | | | | | | | |
| --- | --- | --- | --- | --- | --- | --- | --- | --- | --- | --- | --- | --- | --- | --- | --- | --- | --- | --- | --- | --- |
| Peak no. | **Glycan composition** | **Replicate A** | | **Replicate B** | | **Replicate C** | | **Replicate D** | | **Replicate E** | | **Replicate F** | | **Replicate G** | | **Replicate H** | | **Data** | | |
|  |  | **Area** | **RA (%)** | **Area** | **RA (%)** | **Area** | **RA (%)** | **Area** | **RA (%)** | **Area** | **RA (%)** | **Area** | **RA (%)** | **Area** | **RA (%)** | **Area** | **RA (%)** | **Avg. RA (%)** | **SD** | **CV** |
| 1 | **N2** | 3573 | 0.86 | 1572 | 0.72 | 2546 | 0.64 | 4558 | 0.71 | 3096 | 1.09 | 4154 | 1.09 | 3222 | 0.78 | 3808 | 0.79 | **0.84** | 0.17 | **20.19** |
| 2 | **N1S1** | 67603 | 16.31 | 36740 | 16.92 | 66979 | 16.93 | 102330 | 15.88 | 49883 | 17.55 | 64788 | 16.97 | 66971 | 16.22 | 81042 | 16.88 | **16.71** | 0.53 | **3.19** |
| 3 | **H1N1F1** | 6284 | 1.52 | 3311 | 1.53 | 5902 | 1.49 | 9636 | 1.50 | 4696 | 1.65 | 6654 | 1.74 | 6584 | 1.59 | 7271 | 1.51 | **1.57** | 0.09 | **5.75** |
| 4 | **N1Sg1** | 35110 | 8.47 | 19332 | 8.90 | 28342 | 7.16 | 51355 | 7.97 | 24775 | 8.71 | 32112 | 8.41 | 32496 | 7.87 | 40099 | 8.35 | **8.23** | 0.55 | **6.71** |
| 5 | **H1N2** | 2282 | 0.55 | 1137 | 0.52 | 1919 | 0.49 | 3229 | 0.50 | 1874 | 0.66 | 2410 | 0.63 | 2246 | 0.54 | 2773 | 0.58 | **0.56** | 0.06 | **10.90** |
| 6 | **N3** | 3089 | 0.75 | 1651 | 0.76 | 2499 | 0.63 | 4383 | 0.68 | 2431 | 0.86 | 3233 | 0.85 | 3149 | 0.76 | 3956 | 0.82 | **0.76** | 0.08 | **10.35** |
| 7 | **H1N1S1** | 4927 | 1.19 | 2827 | 1.30 | 4681 | 1.18 | 7449 | 1.16 | 3213 | 1.13 | 4372 | 1.15 | 4988 | 1.21 | 5499 | 1.15 | **1.18** | 0.06 | **4.66** |
| 8 | **H1N1Sg1** | 2357 | 0.57 | 1309 | 0.60 | 1815 | 0.46 | 3363 | 0.52 | 2016 | 0.71 | 2501 | 0.66 | 2365 | 0.57 | 3403 | 0.71 | **0.60** | 0.09 | **14.73** |
| 9 | **N2S1** | 83444 | 20.14 | 46125 | 21.25 | 86691 | 21.91 | 129686 | 20.12 | 58101 | 20.44 | 72354 | 18.95 | 82022 | 19.86 | 102911 | 21.44 | **20.51** | 0.96 | **4.70** |
| 10 | **H1N2F1** | 13959 | 3.37 | 6571 | 3.03 | 12153 | 3.07 | 19938 | 3.09 | 8644 | 3.04 | 12179 | 3.19 | 14037 | 3.40 | 15702 | 3.27 | **3.18** | 0.15 | **4.66** |
| 11 | **N2Sg1** | 48297 | 11.65 | 26433 | 12.18 | 40631 | 10.27 | 74549 | 11.57 | 32654 | 11.49 | 41330 | 10.82 | 45716 | 11.07 | 58659 | 12.22 | **11.41** | 0.66 | **5.82** |
| 12 | **H2N2** | 1928 | 0.47 | 1007 | 0.46 | 1610 | 0.41 | 2591 | 0.40 | 1749 | 0.62 | 2322 | 0.61 | 1721 | 0.42 | 2669 | 0.56 | **0.49** | 0.09 | **18.07** |
| 13 | **H1N3** | 1752 | 0.42 | 744 | 0.34 | 1445 | 0.37 | 2626 | 0.41 | 1421 | 0.50 | 1741 | 0.46 | 1793 | 0.43 | 2191 | 0.46 | **0.42** | 0.05 | **12.07** |
| 14 | **H1N1F1S1** | 3275 | 0.79 | 1744 | 0.80 | 3764 | 0.95 | 5382 | 0.84 | 1987 | 0.70 | 2877 | 0.75 | 3496 | 0.85 | 3647 | 0.76 | **0.80** | 0.08 | **9.42** |
| 15 | **H1N1F1Sg1** | 1788 | 0.43 | 993 | 0.46 | 1637 | 0.41 | 2717 | 0.42 | 1099 | 0.39 | 1473 | 0.39 | 1697 | 0.41 | 1957 | 0.41 | **0.41** | 0.02 | **5.65** |
| 16 | **H1N2F2** | 1362 | 0.33 | 588 | 0.27 | 1112 | 0.28 | 2049 | 0.32 | 636 | 0.22 | 1291 | 0.34 | 1358 | 0.33 | 1203 | 0.25 | **0.29** | 0.04 | **14.42** |
| 17 | **H1N2S1** | 4598 | 0.11 | 2553 | 1.18 | 4795 | 1.21 | 7521 | 1.17 | 2997 | 1.05 | 4110 | 1.08 | 4588 | 1.11 | 5341 | 1.11 | **1.13** | 0.05 | **4.72** |
| 18 | **H2N2F1** | 4034 | 0.97 | 1914 | 0.88 | 3720 | 0.94 | 6333 | 0.98 | 2594 | 0.91 | 4026 | 1.05 | 4243 | 1.03 | 4672 | 0.97 | **0.97** | 0.06 | **5.87** |
| 19 | **H1N2Sg1** | 3065 | 0.74 | 1633 | 0.75 | 2591 | 0.65 | 4884 | 0.76 | 2324 | 0.82 | 3100 | 0.81 | 2994 | 0.73 | 3671 | 0.76 | **0.75** | 0.05 | **6.81** |
| 20 | **H1N3F1** | 8174 | 1.97 | 3728 | 1.72 | 7516 | 1.90 | 13008 | 2.02 | 5155 | 1.81 | 7780 | 2.04 | 8394 | 2.03 | 8709 | 1.81 | **1.91** | 0.12 | **6.33** |
| 21 | **H1N2F2** | 810 | 0.20 | 382 | 0.18 | 826 | 0.21 | 1265 | 0.20 | 739 | 0.26 | 983 | 0.26 | 868 | 0.21 | 1110 | 0.23 | **0.22** | 0.03 | **13.91** |
| 22 | **H1N1S2** | 2245 | 0.54 | 1368 | 0.63 | 2865 | 0.72 | 4084 | 0.63 | 1565 | 0.55 | 2183 | 0.57 | 2458 | 0.60 | 2883 | 0.60 | **0.61** | 0.06 | **9.63** |
| 23 | **H1N1S1Sg1** | 876 | 0.21 | 457 | 0.21 | 1097 | 0.28 | 1465 | 0.23 | 673 | 0.24 | 811 | 0.21 | 919 | 0.22 | 1024 | 0.21 | **0.23** | 0.02 | **9.95** |
| 24 | **H1N2F1S1** | 13362 | 3.22 | 6646 | 3.06 | 13489 | 3.41 | 23739 | 3.68 | 8390 | 2.95 | 12012 | 3.15 | 13915 | 3.37 | 14250 | 2.97 | **3.23** | 0.25 | **7.76** |
| 25 | **H2N2F2** | 17510 | 4.23 | 7781 | 3.58 | 16650 | 4.21 | 30236 | 4.69 | 9814 | 3.45 | 15908 | 4.17 | 17720 | 4.29 | 17008 | 3.54 | **4.02** | 0.44 | **10.98** |
| 26 | **H2N2S1** | 8205 | 1.98 | 4160 | 1.92 | 7162 | 1.81 | 14069 | 2.18 | 5227 | 1.84 | 7673 | 2.01 | 8444 | 2.04 | 8897 | 1.85 | **1.95** | 0.13 | **6.41** |
| 27 | **H3N2F1** | 1394 | 0.34 | 636 | 0.29 | 1513 | 0.38 | 2178 | 0.34 | 1220 | 0.43 | 1600 | 0.42 | 1455 | 0.35 | 1842 | 0.38 | **0.37** | 0.05 | **12.44** |
| 28 | **H1N3F2** | 2285 | 0.55 | 1012 | 0.47 | 2027 | 0.51 | 3858 | 0.60 | 1533 | 0.54 | 2441 | 0.64 | 2509 | 0.61 | 2521 | 0.53 | **0.55** | 0.06 | **10.25** |
| 29 | **H2N3F1** | 2223 | 0.54 | 1079 | 0.50 | 2344 | 0.59 | 3641 | 0.56 | 1541 | 0.54 | 2288 | 0.60 | 2188 | 0.53 | 2347 | 0.49 | **0.54** | 0.04 | **7.40** |
| 30 | **H1N4F1** | 3883 | 0.94 | 1786 | 0.82 | 3446 | 0.87 | 5975 | 0.93 | 2674 | 0.94 | 4007 | 1.05 | 4161 | 1.01 | 3938 | 0.82 | **0.92** | 0.08 | **8.89** |
| 31 | **H1N2F2S1** | 6586 | 1.59 | 3357 | 1.55 | 6460 | 1.63 | 11403 | 1.77 | 4046 | 1.42 | 6085 | 1.59 | 7236 | 1.75 | 7356 | 1.53 | **1.60** | 0.11 | **7.13** |
| 32 | **H2N2F3** | 4512 | 1.09 | 2035 | 0.94 | 4422 | 1.12 | 7854 | 1.22 | 2708 | 0.95 | 4209 | 1.10 | 5093 | 1.23 | 4832 | 1.01 | **1.08** | 0.11 | **10.28** |
| 33 | **H1N2F2Sg1** | 4830 | 1.17 | 2313 | 1.07 | 4280 | 1.08 | 7831 | 1.22 | 3053 | 1.07 | 4447 | 1.16 | 5269 | 1.28 | 5301 | 1.10 | **1.14** | 0.08 | **6.60** |
| 34 | **H2N2F1Sg1** | 2334 | 0.56 | 1079 | 0.50 | 2075 | 0.52 | 3560 | 0.55 | 2260 | 0.79 | 2936 | 0.77 | 2752 | 0.67 | 3271 | 0.68 | **0.63** | 0.11 | **17.95** |
| 35 | **H1N3F1S1** | 7383 | 1.78 | 4025 | 1.85 | 8586 | 2.17 | 12161 | 1.89 | 4750 | 1.67 | 6693 | 1.75 | 7463 | 1.81 | 7494 | 1.56 | **1.81** | 0.18 | **9.86** |
| 36 | **H2N3F2** | 12254 | 2.96 | 5739 | 2.64 | 12668 | 3.20 | 19900 | 3.09 | 7481 | 2.63 | 11707 | 3.07 | 12754 | 3.09 | 12105 | 2.52 | **2.90** | 0.26 | **8.97** |
| 37 | **H1N3F1Sg1** | 4755 | 1.15 | 2598 | 1.20 | 4839 | 1.22 | 7862 | 1.22 | 3227 | 1.14 | 4236 | 1.11 | 4880 | 1.18 | 4923 | 1.03 | **1.15** | 0.07 | **5.72** |
| 38 | **H2N3F3** | 2943 | 0.71 | 1413 | 0.65 | 2965 | 0.75 | 4660 | 0.72 | 1986 | 0.70 | 2858 | 0.75 | 3353 | 0.81 | 3352 | 0.70 | **0.72** | 0.05 | **6.57** |
| 39 | **H3N3S1** | 3313 | 0.80 | 1511 | 0.70 | 2500 | 0.63 | 4379 | 0.68 | 3276 | 1.15 | 3919 | 1.03 | 3721 | 0.90 | 5146 | 1.07 | **0.87** | 0.20 | **22.75** |
| 40 | **H2N4F2** | 11807 | 2.85 | 5803 | 2.67 | 13098 | 3.31 | 16766 | 2.60 | 6772 | 2.38 | 10002 | 2.62 | 11715 | 2.84 | 11273 | 2.35 | **2.70** | 0.31 | **11.32** |

**Table N.**

1. **Intermediate precision (interday variation)**

| MALDI-TOF-MS data of permethylated *O*-glycans released by semi-automated reductive β-elimination (DAY 1) | | | | | | | | | | | | | | | | | | | | |
| --- | --- | --- | --- | --- | --- | --- | --- | --- | --- | --- | --- | --- | --- | --- | --- | --- | --- | --- | --- | --- |
| Peak no. | **Glycan composition** | **Replicate A** | | **Replicate B** | | **Replicate C** | | **Replicate D** | | **Replicate E** | | **Replicate F** | | **Replicate G** | | **Replicate H** | |  | | |
|  |  | **Area** | **RA (%)** | **Area** | **RA (%)** | **Area** | **RA (%)** | **Area** | **RA (%)** | **Area** | **RA (%)** | **Area** | **RA (%)** | **Area** | **RA (%)** | **Area** | **RA (%)** |  |  |  |
| 1 | **N1S1** | 67603 | 22.43 | 36740 | 22.80 | 66979 | 23.08 | 102330 | 22.08 | 49883 | 24.14 | 64788 | 23.91 | 66971 | 22.66 | 81042 | 23.01 |  |  |  |
| 2 | **N1Sg1** | 35110 | 11.65 | 19332 | 12.00 | 28342 | 9.77 | 51355 | 11.08 | 24775 | 11.99 | 32112 | 11.85 | 32496 | 11.00 | 40099 | 11.38 |  |  |  |
| 3 | **N2S1** | 83444 | 27.69 | 46125 | 28.62 | 86691 | 29.88 | 129686 | 27.98 | 58101 | 28.12 | 72354 | 26.70 | 82022 | 27.76 | 102911 | 29.22 |  |  |  |
| 4 | **H1N2F1** | 13959 | 4.63 | 6571 | 4.08 | 12153 | 4.19 | 19938 | 4.30 | 8644 | 4.18 | 12179 | 4.49 | 14037 | 4.75 | 15702 | 4.46 |  |  |  |
| 5 | **N2Sg1** | 48297 | 16.02 | 26433 | 16.40 | 40631 | 14.00 | 74549 | 16.09 | 32654 | 15.81 | 41330 | 15.25 | 45716 | 15.47 | 58659 | 16.65 |  |  |  |
| 6 | **H1N3F1** | 8174 | 2.71 | 3728 | 2.31 | 7516 | 2.59 | 13008 | 2.81 | 5155 | 2.50 | 7780 | 2.87 | 8394 | 2.84 | 8709 | 2.47 |  |  |  |
| 7 | **H1N2F1S1** | 13362 | 4.43 | 6646 | 4.12 | 13489 | 4.65 | 23739 | 5.12 | 8390 | 4.06 | 12012 | 4.43 | 13915 | 4.71 | 14250 | 4.05 |  |  |  |
| 8 | **H1N3F1S1** | 7383 | 2.45 | 4025 | 2.50 | 8586 | 2.96 | 12161 | 2.62 | 4750 | 2.30 | 6693 | 2.47 | 7463 | 2.53 | 7494 | 2.13 |  |  |  |
| 9 | **H2N3F2** | 12254 | 4.07 | 5739 | 3.56 | 12668 | 4.37 | 19900 | 4.29 | 7481 | 3.62 | 11707 | 4.32 | 12754 | 4.32 | 12105 | 3.44 |  |  |  |
| 10 | **H2N4F2** | 11807 | 3.92 | 5803 | 3.60 | 13098 | 4.51 | 16766 | 3.62 | 6772 | 3.28 | 10002 | 3.69 | 11715 | 3.96 | 11273 | 3.20 |  |  |  |

| MALDI-TOF-MS data of permethylated *O*-glycans released by semi-automated reductive β-elimination (DAY 2) | | | | | | | | | | | | | | | | | | | | |
| --- | --- | --- | --- | --- | --- | --- | --- | --- | --- | --- | --- | --- | --- | --- | --- | --- | --- | --- | --- | --- |
| Peak no. | **Glycan composition** | **Replicate A** | | **Replicate B** | | **Replicate C** | | **Replicate D** | | **Replicate E** | | **Replicate F** | | **Replicate G** | | **Replicate H** | | **Data (DAY 1 vs DAY 2)** | | |
|  |  | **Area** | **RA (%)** | **Area** | **RA (%)** | **Area** | **RA (%)** | **Area** | **RA (%)** | **Area** | **RA (%)** | **Area** | **RA (%)** | **Area** | **RA (%)** | **Area** | **RA (%)** | **Avg. RA (%)** | **SD** | **CV** |
| 1 | **N1S1** | 1945 | 26.83 | 6003 | 26.69 | 1024 | 27.97 | 1269 | 25.75 | 19302 | 22.26 | 10545 | 24.36 | 41094 | 24.13 | 113140 | 24.02 | **24.13** | 1.79 | **7.43** |
| 2 | **N1Sg1** | 974 | 13.44 | 3002 | 13.35 | 507 | 13.85 | 568 | 11.53 | 9267 | 10.69 | 5118 | 11.82 | 20662 | 12.13 | 54850 | 11.64 | **11.82** | 1.04 | **8.81** |
| 3 | **N2S1** | 1916 | 26.43 | 5680 | 25.25 | 978 | 26.72 | 1421 | 28.84 | 24320 | 28.05 | 11362 | 26.25 | 43923 | 25.79 | 123231 | 26.16 | **27.47** | 1.33 | **4.83** |
| 4 | **H1N2F1** | 375 | 5.17 | 1165 | 5.18 | 199 | 5.44 | 229 | 4.65 | 4477 | 5.16 | 2278 | 5.26 | 8907 | 5.23 | 21682 | 4.60 | **4.74** | 0.45 | **9.41** |
| 5 | **N2Sg1** | 1045 | 14.42 | 3158 | 14.04 | 521 | 14.23 | 710 | 14.41 | 13567 | 15.65 | 6177 | 14.27 | 25731 | 15.11 | 70746 | 15.02 | **15.18** | 0.88 | **5.77** |
| 6 | **H1N3F1** | 208 | 2.87 | 622 | 2.77 | 87.6 | 2.39 | 115 | 2.33 | 2689 | 3.10 | 1266 | 2.92 | 5331 | 3.13 | 14767 | 3.14 | **2.73** | 0.28 | **10.10** |
| 7 | **H1N2F1S1** | 278 | 3.84 | 956 | 4.25 | 124 | 3.39 | 199 | 4.04 | 4410 | 5.09 | 2104 | 4.86 | 6616 | 3.89 | 23056 | 4.89 | **4.36** | 0.49 | **11.33** |
| 8 | **H1N3F1S1** | 148 | 2.04 | 413 | 1.84 | 69 | 1.88 | 105 | 2.13 | 2021 | 2.33 | 986 | 2.28 | 3784 | 2.22 | 11558 | 2.45 | **2.32** | 0.28 | **12.28** |
| 9 | **H2N3F2** | 209 | 2.88 | 840 | 3.73 | 69.5 | 1.90 | 191 | 3.88 | 3744 | 4.32 | 2013 | 4.65 | 7250 | 4.26 | 21220 | 4.51 | **3.88** | 0.70 | **18.16** |
| 10 | **H2N4F2** | 151 | 2.08 | 652 | 2.90 | 81.6 | 2.23 | 121 | 2.46 | 2915 | 3.36 | 1439 | 3.32 | 6994 | 4.11 | 16771 | 3.56 | **3.36** | 0.67 | **20.04** |

**Table O.**

1. **Linearity**

| Peak no. | Sample amount (μg) | Glycan composition | MALDI-TOF-MS data of permethylated *O*-glycans released by semi-automated reductive β-elimination | | | | | | |
| --- | --- | --- | --- | --- | --- | --- | --- | --- | --- |
|  |  |  | **Replicate A** | | **Replicate B** | | **Replicate C** | | **Avg. RI (%)** |
|  |  |  | **Intensity** | **RI (%)** | **Intensity** | **RI (%)** | **Intensity** | **RI (%)** |  |
| 1 | 5 | **N1S1** | 134761 | **27.21** | 165162 | **27.08** | 151348 | **26.94** | **27.08** |
| 2 |  | **N1Sg1** | 62618 | **12.64** | 80514 | **13.20** | 74427 | **13.25** | **13.03** |
| 3 |  | **N2S1** | 148326 | **29.95** | 179391 | **29.42** | 164684 | **29.32** | **29.56** |
| 4 |  | **H1N2F1** | 22820 | **4.61** | 25212 | **4.13** | 25671 | **4.57** | **4.44** |
| 5 |  | **N2Sg1** | 81676 | **16.49** | 99435 | **16.31** | 90697 | **16.15** | **16.31** |
| 6 |  | **H1N3F1** | 10520 | **2.12** | 13123 | **2.15** | 12523 | **2.23** | **2.17** |
| 7 |  | **H1N2F1S1** | 14659 | **2.96** | 18753 | **3.08** | 16956 | **3.02** | **3.02** |
| 8 |  | **H1N3F1S1** | 5586 | **1.13** | 8016 | **1.31** | 7099 | **1.26** | **1.24** |
| 9 |  | **H2N3F2** | 8419 | **1.70** | 11671 | **1.91** | 10837 | **1.93** | **1.85** |
| 10 |  | **H2N4F2** | 5900 | **1.19** | 8527 | **1.40** | 7511 | **1.34** | **1.31** |
| 1 | 10 | **N1S1** | 284696 | **25.85** | 195283 | **25.89** | 296944 | **29.70** | **27.15** |
| 2 |  | **N1Sg1** | 150409 | **13.66** | 97676 | **12.95** | 112550 | **11.26** | **12.62** |
| 3 |  | **N2S1** | 322809 | **29.31** | 227736 | **30.19** | 310694 | **31.08** | **30.20** |
| 4 |  | **H1N2F1** | 40951 | **3.72** | 31982 | **4.24** | 45003 | **4.50** | **4.15** |
| 5 |  | **N2Sg1** | 188759 | **17.14** | 124431 | **16.50** | 132022 | **13.21** | **15.61** |
| 6 |  | **H1N3F1** | 21928 | **1.99** | 15332 | **2.03** | 20886 | **2.09** | **2.04** |
| 7 |  | **H1N2F1S1** | 36472 | **3.31** | 23588 | **3.13** | 30353 | **3.04** | **3.16** |
| 8 |  | **H1N3F1S1** | 17228 | **1.56** | 11199 | **1.48** | 14543 | **1.45** | **1.50** |
| 9 |  | **H2N3F2** | 22198 | **2.02** | 14871 | **1.97** | 20712 | **2.07** | **2.02** |
| 10 |  | **H2N4F2** | 15783 | **1.43** | 12181 | **1.61** | 15960 | **1.60** | **1.55** |
| 1 | 50 | **N1S1** | 376050 | **27.53** | 263210 | **27.68** | 255822 | **27.94** | **27.72** |
| 2 |  | **N1Sg1** | 176866 | **12.95** | 127211 | **13.38** | 121313 | **13.25** | **13.19** |
| 3 |  | **N2S1** | 399618 | **29.25** | 273366 | **28.75** | 265699 | **29.02** | **29.01** |
| 4 |  | **H1N2F1** | 56958 | **4.17** | 35963 | **3.78** | 37575 | **4.10** | **4.02** |
| 5 |  | **N2Sg1** | 210497 | **15.41** | 148442 | **15.61** | 142162 | **15.53** | **15.52** |
| 6 |  | **H1N3F1** | 35345 | **2.59** | 21584 | **2.27** | 21294 | **2.33** | **2.39** |
| 7 |  | **H1N2F1S1** | 44840 | **3.28** | 31037 | **3.26** | 27790 | **3.04** | **3.19** |
| 8 |  | **H1N3F1S1** | 17938 | **1.31** | 13539 | **1.42** | 12680 | **1.38** | **1.37** |
| 9 |  | **H2N3F2** | 27728 | **2.03** | 21010 | **2.21** | 17258 | **1.88** | **2.04** |
| 10 |  | **H2N4F2** | 20197 | **1.48** | 15391 | **1.62** | 14042 | **1.53** | **1.54** |
| 1 | 100 | **N1S1** | 200395 | **27.72** | 276334 | **30.39** | 285352 | **28.55** | **28.89** |
| 2 |  | **N1Sg1** | 93728 | **12.97** | 128010 | **14.08** | 132718 | **13.28** | **13.44** |
| 3 |  | **N2S1** | 212318 | **29.37** | 248364 | **27.31** | 284795 | **28.49** | **28.39** |
| 4 |  | **H1N2F1** | 33724 | **4.66** | 35451 | **3.90** | 40359 | **4.04** | **4.20** |
| 5 |  | **N2Sg1** | 110158 | **15.24** | 132526 | **14.57** | 151320 | **15.14** | **14.98** |
| 6 |  | **H1N3F1** | 15027 | **2.08** | 17306 | **1.90** | 20130 | **2.01** | **2.00** |
| 7 |  | **H1N2F1S1** | 22436 | **3.10** | 27637 | **3.04** | 33104 | **3.31** | **3.15** |
| 8 |  | **H1N3F1S1** | 9438 | **1.31** | 12434 | **1.37** | 15064 | **1.51** | **1.39** |
| 9 |  | **H2N3F2** | 14343 | **1.98** | 17961 | **1.98** | 21087 | **2.11** | **2.02** |
| 10 |  | **H2N4F2** | 11363 | **1.57** | 13247 | **1.46** | 15666 | **1.57** | **1.53** |
| 1 | 200 | **N1S1** | 3291 | **32.04** | 10232 | **31.90** | 4495 | **31.28** | **31.74** |
| 2 |  | **N1Sg1** | 1665 | **16.21** | 4906 | **15.30** | 1851 | **12.88** | **14.80** |
| 3 |  | **N2S1** | 2519 | **24.52** | 8137 | **25.37** | 3774 | **26.27** | **25.39** |
| 4 |  | **H1N2F1** | 460 | **4.48** | 1446 | **4.51** | 652 | **4.54** | **4.51** |
| 5 |  | **N2Sg1** | 1468 | **14.29** | 4544 | **14.17** | 2029 | **14.12** | **14.19** |
| 6 |  | **H1N3F1** | 205 | **2.00** | 516 | **1.61** | 287 | **2.00** | **1.87** |
| 7 |  | **H1N2F1S1** | 273 | **2.66** | 910 | **2.84** | 487 | **3.39** | **2.96** |
| 8 |  | **H1N3F1S1** | 99.6 | **0.97** | 374 | **1.17** | 257 | **1.79** | **1.31** |
| 9 |  | **H2N3F2** | 196 | **1.91** | 639 | **1.99** | 308 | **2.14** | **2.01** |
| 10 |  | **H2N4F2** | 95.7 | **0.93** | 368 | **1.15** | 228 | **1.59** | **1.22** |

**Table P.**

**References**

1. Jeong HJ, Adhya M, Park HM, Kim YG, Kim BG. Detection of Hanganutziu-Deicher antigens in O-glycans from pig heart tissues by matrix-assisted laser desorption/ionization time-of-flight mass spectrometry. Xenotransplantation. 2013;20(6):407–17.

2. Kozak RP, Royle L, Gardner RA, Fernandes DL, Wuhrer M. Suppression of peeling during the release of O-glycans by hydrazinolysis. Anal Biochem [Internet]. Elsevier Inc.; 2012;423(1):119–28. Available from: http://dx.doi.org/10.1016/j.ab.2012.01.002

3. Ich. ICH Topic Q2 (R1) Validation of Analytical Procedures : Text and Methodology. Int Conf Harmon. 2005;1994(November 1996):17.
